# Supplementary material for: Phytochemical characterization, anticancer potential, and nanoemulsion-based delivery of Chiliadenus montanus
Source: PLoS One. 2025 Sep 26;20(9):e0333482. doi: 10.1371/journal.pone.0333482 (PMC12469210; doi:10.1371/journal.pone.0333482)
Supplement: Attachment — Submitted filename: PONE-D-25-17902_R1_reviewer.pdf [file pone.0333482.s002.pdf]

PLOS ONE

Phytochemical Characterization, Anticancer Potential, and Nanoemulsion-Based Delivery of Chiliadenus montanus

--Manuscript Draft--

**Manuscript Number:**	PONE-D-25-17902R1
**Article Type:**	Research Article
**Full Title:**	Phytochemical Characterization, Anticancer Potential, and Nanoemulsion-Based Delivery of Chiliadenus montanus
**Short Title:**	Phytochemical Characterization, Anticancer Potential, and Nanoemulsion-Based Delivery of Chiliadenus montanus
**Corresponding Author:**	NOUR Aboalhaija<br>Al-Zaytoonah University of Jordan Faculty of Pharmacy<br>Amman, JORDAN
**Keywords:**	Chiliadenus montanus; Anticancer; nanoemulsion; pulmonary delivery; LC-MS; SPME; GC-MS
**Abstract:**	Chiliadenus montanus (Vahl) Boiss. (Asteraceae) is a pharmacologically significant plant with different potent pharmacological properties. This study aimed to evaluate its phytochemical and biological properties and develop nanoemulsions (NEs) to enhance pulmonary delivery for lung carcinoma treatment. Ethanol and water extracts, along with petroleum ether, chloroform, ethyl acetate, and methanol fractions, were assessed for total phenol and flavonoid contents, antioxidant activity, and cytotoxicity against H1299 and A549 lung cancer cell lines. Ethyl acetate fraction exhibited the highest phenol (47.94 ± 0.32 mg GAE/g) and flavonoid (20.34 ± 1.48 mg rutin/g) contents, while the ethanol extract showed the strongest antioxidant activity (IC_{50} = 322.1 µg/mL) and selective cytotoxicity (IC_{50} = 641.2 µg/mL) against H1299 cells. Nevadensin, chlorogenic acid, and sorbifolin were identified as the major constituents of the ethanol extract using liquid chromatography-mass spectrometry (LC-MS) analysis. Gas chromatography-mass spectrometry (GC-MS) analysis revealed α-phellandrene, 1,8-cineole, and α-cadinol as the major volatile constituents. The major volatile compound of the aroma profile of the aerial parts, determined by solid phase micro extraction (SPME) was 1,8-cineole. Spontaneous emulsification was used to formulate ethanolic NEs, with S3, S4, S5, and S6 demonstrating thermodynamic stability, suitable pulmonary pH, and droplet sizes below 200 nm. These findings highlight the promising potential of C. montanus NEs as stable pulmonary drug delivery systems for lung carcinoma therapy.
**Order of Authors:**	NOUR Aboalhaija
	Hala Abulawi
	Rania Hamed
	Mohammad Alwahsh
	Fatma Afifi
	Heba Syaj
	Elham Abusharieh
	Ismail Abaza
**Response to Reviewers:**	Re: Manuscript ID: PONE-D-25-17902<br><br>Dear Dr. Šiler,<br>We sincerely thank you and the reviewers for your constructive and insightful comments on our manuscript. We appreciate the opportunity to revise and resubmit our work.<br>We have carefully considered each comment and revised the manuscript accordingly. In brief:<br>1.Please update the Latin name of the study species. Varhemia montana is actually a

	synonym of Chiliadenus montanus (Vahl) Brullo (e.g., https://powo.science.kew.org/taxon/urn:lsid:ipni.org:names:257204-1).<br>The Latin name of the plant species has been updated to Chiliadenus montanus (Vahl) Brullo, in accordance with current taxonomic classifications.<br><br>2.The whole first paragraph of the introductory section needs thorough revision, as the taxonomy of the former genus Varhemia has been changed since 2018.<br>The first paragraph of the introduction has been thoroughly revised to reflect this change.<br>3.Please add line and page numbers to facilitate the future review process.<br>Line and page numbers have been added throughout the manuscript to aid future review.<br>4.Please explain letters in superscripts in Table 2.<br>The superscript letters in Table 2 have been clearly explained in a new footnote to enhance clarity.<br>5.Figure Reorganization: As per PLOS ONE's figure formatting guidelines.<br>We have uploaded updated versions of all figures. Figure 1a–c has been split into three separate figures to meet the requirements. We have also split Figure 4 as necessary and uploaded the revised versions accordingly.<br><br>We hope the revised version meets your expectations and the requirements of PLOS ONE. Thank you again for your time and consideration.<br><br>Nour Aboalhaija, PhD<br>Assistant Professor of Phytochemistry<br>Department of Pharmacy<br>Faculty of Pharmacy<br>Al-Zaytoonah University of Jordan<br>Mobile: +962-79-6304081<br>E-mail: n.aboalhaija@zuj.edu.jo<br>Google Scholar: https://scholar.google.com/citations?user=CBVZdtcAAAAJ&hl=en<br>ResearchGate: https://www.researchgate.net/profile/Nour-Aboalhaija
**Additional Information:**	
**Question**	**Response**
**Financial Disclosure**<br><br>Enter a financial disclosure statement that describes the sources of funding for the work included in this submission. Review the submission guidelines for detailed requirements. View published research articles from *PLOS ONE* for specific examples.<br><br>This statement is required for submission and **will appear in the published article** if the submission is accepted. Please make sure it is accurate.	Yes

Powered by Editorial Manager® and ProduXion Manager® from Aries Systems Corporation

**Funded studies**<br>Enter a statement with the following details:<br>• Initials of the authors who received each award<br>• Grant numbers awarded to each author<br>• The full name of each funder<br>• URL of each funder website<br>• Did the sponsors or funders play any role in the study design, data collection and analysis, decision to publish, or preparation of the manuscript?<br><br>Did you receive funding for this work?	
Please add funding details.<br>as follow-up to "**Financial Disclosure**<br><br>Enter a financial disclosure statement that describes the sources of funding for the work included in this submission. Review the submission guidelines for detailed requirements. View published research articles from *PLOS ONE* for specific examples.<br><br>This statement is required for submission and **will appear in the published article** if the submission is accepted. Please make sure it is accurate.<br><br>**Funded studies**<br>Enter a statement with the following details:<br>• Initials of the authors who received each award<br>• Grant numbers awarded to each author<br>• The full name of each funder<br>• URL of each funder website<br>• Did the sponsors or funders play any role in the study design, data collection and analysis, decision to publish, or preparation of the manuscript?<br><br>Did you receive funding for this work?"	This research was funded by Deanship of Scientific Research and Innovation at Al-Zaytoonah University of Jordan (2022-2023/07/23).
Please select the country of your main research funder (please select carefully as in some cases this is used in fee calculation).	JORDAN - JO

as follow-up to "**Financial Disclosure**<br><br>Enter a financial disclosure statement that describes the sources of funding for the work included in this submission. Review the submission guidelines for detailed requirements. View published research articles from *PLOS ONE* for specific examples.<br><br>This statement is required for submission and **will appear in the published article** if the submission is accepted. Please make sure it is accurate.<br><br>**Funded studies**<br>Enter a statement with the following details:<br>• Initials of the authors who received each award<br>• Grant numbers awarded to each author<br>• The full name of each funder<br>• URL of each funder website<br>• Did the sponsors or funders play any role in the study design, data collection and analysis, decision to publish, or preparation of the manuscript?<br><br>Did you receive funding for this work?"	
**Competing Interests**<br><br>Use the instructions below to enter a competing interest statement for this submission. On behalf of all authors, disclose any competing interests that could be perceived to bias this work—acknowledging all financial support and any other relevant financial or non-financial competing interests.<br><br>This statement is required for submission and **will appear in the published article** if the submission is accepted. Please make sure it is accurate and that any funding sources listed in your Funding Information later in the submission form are also declared in your Financial Disclosure statement.	The authors declare no conflicts of interest. The funders had no role in the design of the study; in the collection, analyses, or interpretation of data; in the writing of the manuscript; or in the decision to publish the results.

View published research articles from *PLOS ONE* for specific examples.<br><br>**NO authors have competing interests**<br><br>Enter: *The authors have declared that no competing interests exist.*<br><br>**Authors with competing interests**<br><br>Enter competing interest details beginning with this statement:<br><br>*I have read the journal's policy and the authors of this manuscript have the following competing interests: [insert competing interests here]*<br><br>* typeset	
**Ethics Statement**<br><br>Enter an ethics statement for this submission. This statement is required if the study involved:<br><br>• Human participants<br>• Human specimens or tissue<br>• Vertebrate animals or cephalopods<br>• Vertebrate embryos or tissues<br>• Field research<br><br>Write "N/A" if the submission does not require an ethics statement.<br><br>General guidance is provided below. Consult the submission guidelines for detailed instructions. **Make sure that all information entered here is included in the Methods section of the manuscript.**	N/A

**Format for specific study types**<br><br>**Human Subject Research (involving human participants and/or tissue)**<br>• Give the name of the institutional review board or ethics committee that approved the study<br>• Include the approval number and/or a statement indicating approval of this research<br>• Indicate the form of consent obtained (written/oral) or the reason that consent was not obtained (e.g. the data were analyzed anonymously)<br><br>**Animal Research (involving vertebrate animals, embryos or tissues)**<br>• Provide the name of the Institutional Animal Care and Use Committee (IACUC) or other relevant ethics board that reviewed the study protocol, and indicate whether they approved this research or granted a formal waiver of ethical approval<br>• Include an approval number if one was obtained<br>• If the study involved *non-human primates*, add *additional details* about animal welfare and steps taken to ameliorate suffering<br>• If anesthesia, euthanasia, or any kind of animal sacrifice is part of the study, include briefly which substances and/or methods were applied<br><br>**Field Research**<br><br>Include the following details if this study involves the collection of plant, animal, or other materials from a natural setting:<br>• Field permit number<br>• Name of the institution or relevant body that granted permission	
**Data Availability**<br><br>Authors are required to make all data underlying the findings described fully available, without restriction, and from the time of publication. PLOS allows rare exceptions to address legal and ethical concerns. See the PLOS Data Policy and FAQ for detailed information.	Yes - all data are fully available without restriction

A Data Availability Statement describing where the data can be found is required at submission. Your answers to this question constitute the Data Availability Statement and **will be published in the article**, if accepted.<br><br>**Important:** Stating 'data available on request from the author' is not sufficient. If your data are only available upon request, select 'No' for the first question and explain your exceptional situation in the text box.<br><br>Do the authors confirm that all data underlying the findings described in their manuscript are fully available without restriction?	
**Describe where the data may be found in full sentences. If you are copying our sample text, replace any instances of XXX with the appropriate details.**<br><br>• If the data are **held or will be held in a public repository**, include URLs, accession numbers or DOIs. If this information will only be available after acceptance, indicate this by ticking the box below. For example: *All XXX files are available from the XXX database (accession number(s) XXX, XXX.)*.<br>• If the data are all contained **within the manuscript and/or Supporting Information files**, enter the following: *All relevant data are within the manuscript and its Supporting Information files.*<br>• If neither of these applies but you are able to provide **details of access elsewhere**, with or without limitations, please do so. For example:<br><br>*Data cannot be shared publicly because of [XXX]. Data are available from the XXX Institutional Data Access / Ethics Committee (contact via XXX) for researchers who meet the criteria for access to confidential data.*<br><br>*The data underlying the results presented in the study are available from (include the name of the third party*	All relevant data are within the manuscript and its Supporting Information files.

Powered by Editorial Manager® and ProduXion Manager® from Aries Systems Corporation

and contact information or URL).<br>• This text is appropriate if the data are owned by a third party and authors do not have permission to share the data.<br><br>* typeset	
Additional data availability information:	

Powered by Editorial Manager® and ProduXion Manager® from Aries Systems Corporation

Manuscript

Click here to access/download;Manuscript;Manuscript.docx

Phytochemical Characterization, Anticancer Potential, and Nanoemulsion-Based Delivery of *Chiliadenus montanus*

Nour Aboalhaija [1,*], Hala Abulawi [1], Rania Hamed [1], Mohammad Alwahsh [1], Fatma Afifi [2], Heba Syaj [1], Elham Abusharieh [1] and Ismail Abaza [2]

[1] Department of Pharmaceutical Sciences, Faculty of Pharmacy, Al-Zaytoonah University of Jorda[illegible] Amm[illegible]733, Jordan

[2] Department of Pharmaceutical Sciences, School of Pharmacy, The University of Jordan, 11942 Amman, Jordan

* Correspondence: n.aboalhaija@zuj.edu.jo

Abstract: *Chiliadenus montanus* (Vahl) Boiss. (Asteraceae) is a pharmacologically significant plant with different potent pharmacological properties. This study aimed to evaluate its phytochemical and biological properties and develop nanoemulsions (NEs) to enhance pulmonary delivery for lung carcinoma treatment[illegible]anol and water extracts, along with petroleum ether, chloroform, ethyl acetate, and methanol fractions, [illegible]re assessed for total phenol and flavonoid contents, antioxidant activity, and cytotoxicity against H1299 and A549 lung cancer cell line[illegible]thyl acetate fraction exhibited the highest phenol (47.94 ± 0.32 mg GA[illegible] and flavonoid (20.34 ± 1.48 mg ruti[illegible]) contents, while the ethanol extract showed the strongest ant[illegible]dant activity (IC_{50} = 322.1 μg/mL) an[illegible]elective cytotoxicity (IC_{50} = 641.2 μg/mL) against H1299 cells. Nevadensin, chlorogenic acid, and sorbifolin were identified as the major constituents of the ethanol extract using liquid chromatography-mass spectrometry (LC-MS) analysis. Gas chromatography-mass spectrometry (GC-MS) analysis revealed α-phellandrene, 1,8-cineole, and α-cadinol as the major volatile constituents. The major volatile compound of the aroma profile of the aerial parts, determined by solid phase micro extraction (SPME) was 1,8-cineole. Spontaneous emulsification was used to formulate ethanolic NEs, with S3, S4, S5, and S6 demonstrating thermodynamic stability, suitable pulmonary pH, and droplet sizes below 200 nm. These findings highlight the promising potential of *C. montanus* NEs as stable pulmonary drug delivery systems for lung carcinoma therapy.

Keywords: *Chiliadenus montanus* ; anticancer; nanoemulsion; pulmonary delivery; LC-MS; SPME; GC-MS

1. Introduction

The genus *Chiliadenus* (Asteraceae, formerly Compositae) includes three species native in Jordan: *C. montanus* (Vahl) Brullo (syn. *Varthemia montana* Vahl (Boiss.)), *C. candicans* (Delile) Brullo (syn. *V. candicans* Delile (Boiss.)), and *C. iphionoides* (Boiss. & Blanche) Brullo (syn. *V. iphionoides* Boiss. & Blanche[illegible]). Among these, *C. montanus*, commonly known as desert Varthemia, is a perennial bushy herb characteri[illegible] by small leaves, numerous branches, and aromatic, hairy, and sticky stems, reaching a height of 20–50 cm [illegible] It thrives in rocky and semi-arid regions, blooming between September and December with disti[illegible]ve tubular yellow flowers (2). Various species formerly assigned to *Varthemia* have demonstrated significant

pharmacological activities, including antibacterial, antifungal, antioxidant, anti-inflammatory, anticancer, antispasmodic, and antiplatelet effects (2-5).

Phytochemically, *C. montanus* is known to be rich in volatile oils, in volatile and non-volatile terpenoids and in flavonoids, -mainly methoxylated-. All these secondary metabolites are associated with a wide range of biological activities, including antibacterial, antifungal, antioxidant, anti-inflammatory, anticancer, antispasmodic, antiplatelet and hypoglycemic effects (2, 6-12)

The species' possible benefits in treatment of Alzheimer's disease are suggested by El-Bsoumy et al. (10). Several researchers have also emphasized the different traditional uses of *C. montanus* including its benefits in management of diarrhea, kidney problems, stomachaches, and chest ailments, especially recommended in Mediterranean countries (2, 12, 13). Keeping in mind that environmental factors can significantly influence the phytochemical profiles of medicinal plants, further investigations of their extracts is warranted to better understand its traditional uses.

This present study aimed to evaluate the chemical composition and biological activities of extracts and fractions of *C. montanus* growing in Jordan. Their total phenol and flavonoid contents were analyzed, along with their antioxidant and antiproliferative activities. To enhance and expand its phytochemical and biological activities, pharmaceutical formulation of its ethanol extract was approached. The ethanol extract of *C. montanus* was incorporated into a NE formulation and assessed as a potential drug delivery system for lung cancer therapy. By investigating its cytotoxic potential and suitability for pulmonary delivery, this research highlights the therapeutic value of *C. montanus* and its potential role as adjunctive phytotherapy for conventional chemotherapy. The composition of the active extract was evaluated by LC-MS. Additionally, volatile oil composition of the hydro-distilled (HD) essential oil and aroma profile of the aerial parts of this species were determined to obtain a complete picture of its phytochemical composition.

2. Materials and Methods

2.1. Plant Material

Chiliadenus montanus was collected from Balqa region (32.037° N, 35.7288° E) of Jordan, in late September 2022. The plant was authenticated by one of the authors using descriptive references and in comparison, with the herbarium specimen in the School of Pharmacy, The University of Jordan, Amman. Voucher specimen were kept in the School of Pharmacy (FMJ-AST95-3) *C. montanus* is characterized by its multiple branches, hairy appearance, sticky stems, and bushy perennial form with small leaves and a woody base, typically ranging in length from 20 to 50 cm (14).

2.2. Essential Oil Extraction by hydro-distillation and Aroma Profile Determination by Solid-Phase Micro-Extraction (SPME)

For the isolation of the essential oil of *C. montanus* aerial parts of the plant were subjected to hydro-distillation in a Clevenger apparatus for 3 hours. The resulting oil was pooled, dried over anhydrous sodium sulfate (Na_2SO_4) and stored at 4 ºC in amber glass vials until analysis (15). For aroma profiling, solid-phase micro-extraction (SPME) was employed to capture and analyze the volatile compounds as described earlier (16).

2.3. Gas Chromatography-Mass Spectrometry (GC-MS)

The volatile organic compounds emitted from *C. montanus* essential oil were analyzed using GC-MS (16). Compound identification was performed using internal digital libraries, including those from NIST and Wiley, by comparing the acquired retention indices (RI) with published values for C_8-C_{20} *n*-alkanes, measured under similar conditions (17).

The experimental RI values were calculated using the following formula:

$$RI=100\ (n + (N - n)\ x\ (Log\ tr\ (unknown) - logtr\ (n))\ /\ logtr(N) - logtr(n)$$

where RI represents the retention time measured from the time of the unretained small molecule, and *n* and *N* represent the numbers of carbon atoms in the smaller and larger alkanes, respectively. A Hewlett-Packard HP-8590 gas chromatograph equipped with a split-spitless injector (split ratio 1:50) and a flame ionization detector (FID) were utilized for quantitative analysis (% area). The concentration of the identified compounds was determined by measuring the relative peak areas of the oil components.

2.5. Extracts Preparation

In this study, the ethanol extract of *C. montanus* was prepared by soaking the dried and crushed aerial parts in 70% ethanol for three weeks at room temperature (RT). Following this period, ethanol was evaporated under vacuum using a rotary evaporator set at 40 °C. The crude extract was first subjected to defatting by liquid-liquid extraction using petroleum ether in a separatory funnel to remove chlorophyll and fatty materials. This process was repeated until the petroleum ether layer became colorless, ensuring the complete removal of non-polar impurities. The defatted aqueous methanol layers were collected and subsequently fractionated based on polarity using a sequential liquid-liquid extraction approach. Chloroform was employed to isolate non-polar constituents, followed by ethyl acetate to extract intermediately polar phytochemicals. The remaining aqueous methanol layer was designated as the methanol fraction, containing the highly polar components. Additionally, aqueous and ethanol extracts were prepared by soaking the plant material in water and 70% ethanol, respectively, without further fractionation.

2.6. Determination of Total Phenol (TPC) and Total Flavonoid Contents (TFC)

The TPC of *C. montanus* extracts was determined using the Folin-Ciocalteu method. Gallic acid in methanol served as a standard, and five serial dilutions (100, 50, 25, 12.5, and 6.125 µg/mL) were prepared. Absorbance readings were taken at 765 nm in triplicate, with methanol used as a blank. The total phenol content was expressed as gallic acid equivalents (GAE) (18).

To determine TFC, aluminum chloride was utilized. After incubation for 30 min at RT, the absorbance was measured at 415 nm. Rutin served as the calibration standard, with five concentrations (100, 50, 25, 12.5, and 6.125 µg/mL) prepared in methanol. Experiments were carried out in triplicate, and the results were averaged (18).

2.7. Antioxidant Activity

To prepare the DPPH reagent, approximately 4 mg of DPPH powder was dissolved in 100 mL of methanol in an Erlenmeyer flask. The mixture was agitated using a magnetic stirrer at 60 to 100 rpm for 5 min to ensure complete dissolution. To protect the DPPH solution from light and evaporation, the flask was

wrapped in foil and sealed. Ascorbic acid was used as a standard to establish a calibration curve. Ten different concentrations were prepared (1000, 500, 250, 125, 62.5, 31.25, 15.625, 7.815, 3.91, and 1.955 μg/mL). In a test tube, 3 mL of the DPPH solution was mixed with 2 mL of each extract. The reaction was allowed to proceed for 30 min at RT in complete darkness.

After the incubation period, the absorbance of each extract was measured at 517 nm. The percentage of inhibition was calculated using the following formula:

$$I\ \% = (A^\circ - A\varkappa) / A^\circ \times 100$$

where A° represents the absorbance of the control (DPPH solution without extract), and Aϰ is the absorbance of the extract at a specific concentration. The IC_{50} value was determined as the concentration required to inhibit 50% of the DPPH radicals.

2.8. In Vitro Cytotoxicity on Cancer Cell Lines

Human cancer cell lines A549, H1299, and normal fibroblasts were obtained from the American Type Culture Collection (ATCC, Manassas, VA, USA). A549 (CCL-185) and H1299 (CRL-5803) cells were cultured in RPMI-1640 medium, while fibroblasts (PCS-201-018) were cultured in Dulbecco's Modified Eagle's Medium (DMEM). Both media were supplemented with 10% fetal bovine serum (Cytevia, Austria), 1% L-glutamine, penicillin-streptomycin, and HEPES buffer (EuroClone S.P.A., Italy). Cells were incubated at 37 °C in a humidified 5% CO_2 atmosphere and sub-cultured every 2–3 days, with passages limited to 20 (19) Cells were seeded into 96-well plates (10,000 cells/100 μL/well) (SPL Life Sciences Co., Korea). After reaching 70% confluence, cells were treated with serially diluted *C. montanus* extract (2000, 1000, 500, 250, and 125 μM) in five replicates. After 48 hours of incubation, media were replaced with fresh media containing 15 μL of MTT (3-[4,5-dimethyl-thiazol-2-yl]-2,5-diphenyl tetrazolium bromide; ATCC® 30-1010 K) and incubated for 3 hours. Formazan crystals were dissolved with solubilization solution, and absorbance was measured at 570 nm using an HTX multi-mode microplate reader (BioTek, USA). Data were analyzed using GraphPad Prism 8.0.2.

2.9. LC-MS Analysis

Extracts were dissolved in solvent and chromatographic separation was achieved using Burker Daltonik (Bremen, Germany) impact II ESI-Q-TOF system set up with Burker Dalotonik Elute UPLC system. Eluted compounds were ionized in a mass spectrometer, analyzed by mass-to-charge ratio (m/z), and identified based on spectral libraries (20).

2.10. Preparation of C. montanus *Nanoemulsions (NEs)*

The ethanol extract was selected for NE formulation to enhance pulmonary delivery based on the high TPC and TFC of this extract as well as its strong antioxidant and antiproliferative activities. This extract was incorporated into an oil-in-water (O/W) NE using the spontaneous emulsification method (21). Briefly, 100 mg of the extract was dissolved in 1 mL of ethanol, and volumes of either 250 μL or 500 μL of this solution were mixed with 2.25 mL or 1.75 mL of ethanol under continuous stirring for 6 minutes at RT. Oleic acid and Tween 20 were then added to the mixture, followed by the dropwise addition of deionized water to facilitate the formation of the NE. The detailed composition of the prepared formulations is provided in Table 1. The resulting NEs were sonicated for 30 minutes to ensure transparency and remove any bubbles.

The formulations were stored at room temperature (RT) or 4 °C for further stability evaluation. This method allowed for the preparation of stable NEs with varying compositions, which were subsequently characterized for their physicochemical properties and stability. The S4 NE was subjected to thermodynamic stability studies (22).

Table 1. The composition of *C. montanus* NEs prepared using the spontaneous emulsification method.

Formulations	Ethanol Extract (μg)	Ethanol (mL)	Tween20 (mL)	Oleic acid (mL)	Deionized water (mL)	Storage condition
**S1**	500	1.75	1.25	0.5	1.00	RT
**S2**	250	2.25	1.50	0.5	0.75	4°C
**S3**	250	2.25	1.25	0.5	1.00	4°C
**S4**	250	2.25	1.25	0.25	1.25	RT
**S5**	250	2.25	1.25	0.5	1.00	RT
**S6**	250	2.25	1.25	0.25	1.25	4°C

2.11. Characterization of C. montanus NEs

2.11.1. Thermodynamic Stability

Thermodynamic stability was assessed using centrifugation (3500 rpm, 15 min) and heating-cooling cycles (six cycles at 45 °C and 4 °C over 48 hours). Stability was further evaluated with freeze-thaw cycles (-21 °C to 25 °C) over 48 hours.

2.11.2. Physical Stability

Physical stability of NEs was monitored for 60 days at RT and at 4 °C.

2.11.3. pH- and Viscosity Measurements

pH was measured using a Jenway Benchtop pH Meter (Cole-Parmer Ltd, Model 3510, UK). Viscosity was determined with a rotational rheometer (QC Rheolab, Anton Paar, Austria) at 25 °C, with shear rates ranging from 10 to 1000 s^{-1}.

2.11.4. Size Distribution and Zeta Potential

Mean droplet size (MDS), polydispersity index (PDI), and zeta potential (ZP) were measured using dynamic light scattering (DLS) (Nicomp Nano Z3000, Entegris, USA) at 25 °C. Measurements were conducted in triplicate weekly for two months.

2.11.5. Transmission Electron Microscopy (TEM) Analysis

NE morphology was analyzed using a transmission electron microscope (TEM, FEI Morgani 268, Holland). Samples were placed on carbon-coated copper grids, dried for 30 min, and imaged at an accelerating voltage of 120 kV.

3. Results

3.1. Gas Chromatography-Mass Spectrometry (GC-MS) Analysis of C. montanus

The hydro-distillation of *C. montanus* yielded a bright yellow essential oil with an intensive characteristic odor. The yield was 0.094% (v/w). GC-MS analysis revealed 31 constituents, with monoterpenes as the dominant chemical class (74.6% of the total composition). Monoterpenes were further categorized into hydrocarbon monoterpenes (37.7%) and oxygenated monoterpenes (36.9%) as shown in Table 2. Additionally, oxygenated sesquiterpenes contributed to 14.9%, while miscellaneous compounds accounted for 7.5% of the composition. The most abundant compounds identified in the HD extract were α-phellandrene (28.60%), 1,8-cineole (23.30%), and α-cadinol (11.90%).

Solid-phase micro-extraction (SPME) analysis revealed a higher proportion of monoterpenes (93.0%), distributed as hydrocarbon monoterpenes (50.4%) and oxygenated monoterpenes (42.6%). Sesquiterpenes were detected in significantly lower quantities (1.4%), with hydrocarbon sesquiterpenes comprising 0.6% and oxygenated sesquiterpenes 0.8%. The predominant volatile constituents of the aroma profile were 1,8-cineole (35.60%), sabinene (12.70%), and α-artemisia triene (11.40%).

Table 2. Composition of the volatile oils of the fresh aerial parts of *C. montanus* obtained by HD and SPME

No.	KI Lit.*	KI Exp.	Compound	HD %	SPME %
1	923	909	α-Artemisia triene[a]	-	11.40
2	932	933	α- Pinene[a]	00.10	0.40
3	945	965	α- Fenchen[a]	00.20	0.40
4	964	941	Camphene[a]	-	10.00
5	969	1019	Sabinene[a]	-	12.70
6	974	1005	β- Pinene[a]	00.20	5.20
7	988	1016	Myrcene[a]	-	0.10
8	999	1220	Yomogi alcohol[b]	06.00	0.30
9	1002	1063	α-Phellandrene[a]	28.60	0.10
10	1008	1052	Carene[a]	03.00	0.80
11	1014	1181	α-Terpinene[a]	00.90	1.80
12	1020	1172	β- Cymene[a]	00.50	2.20
13	1024	8617	Limonine[e]	-	3.60
14	1025	1031	β-Phellandrene[a]	-	2.10
15	1026	1198	1.8 Cineole[b]	23.30	35.00
16	1034	1225	Santolina alcohol[b]	00.20	2.60

17	1054	1058	Terpinene <γ[a]	-	3.20
18	1065	1252	Sabinene hydrate[b]	00.80	3.80
19	1086	1204	Terpinolene[a]	03.40	-
20	1095	1301	Linalool[b]	01.10	0.10
21	1135	1602	E-Pinocarveol[b]	00.60	0.60
22	1136	1304	E-*p*-Menthenol[e]	01.70	-
23	1141	999	Camphor[b]	00.90	-
24	1160	1378	Pinocarvone[b]	02.70	0.10
25	1165	1665	Borneol[b]	00.50	-
26	1186	1641	α- Terpineol[b]	01.20	0.10
27	1194	1670	Myrtenol[b]	00.20	-
28	1254	1412	Bornyl acetate[e]	00.20	-
29	1277	1641	Ethyl chrysanthemumate[e]	00.20	-
30	1288	1484	Lavandulyl acetate[e]	01.70	-
31	1298	1698	Carvacrol[b]	00.20	-
32	1311	1536	Pinocarvyl acetate[e]	00.10	-
33	1439	1403	Aromadendrene[c]	-	0.30
34	1522	1043	Cadinene[c]	-	0.30
35	1548	1653	α-Cadinol[d]	11.90	0.40
36	1582	1495	Caryophyllene oxide[d]	01.00	0.40
37	1644	1687	α-Muurolol[d]	00.80	-
38	1665	1705	Intermedeol[d]	01.20	-
39	1783	1677	Eudesmol acetate[e]	03.60	-
	**Monoterpenes**			74.6%	93.0%
	Oxygenated monoterpenes[a]			37.7 %	42.6%
	Monoterpene hydrocarbons[b]			36.9%	50.4%
	**Sesquiterpenes**			14.9%	1.4%
	Oxygenated sesquiterpenes[c]			14.9%	0.8%
	Sesquiterpene hydrocarbons[d]			0%	0.6%
	[e]Miscellaneous			07.5%	3.6%
	Total identified			97.0%	98.0%

*Lit KI: Reported Kovats Index, [a]Oxygenated monoterpenes, [b]Monoterpene hydrocarbons, [c]Oxygenated sesquiterpenes, and [d]Sesquiterpene hydrocarbons.

3.2. Total Phenol (TPC) and Total Flavonoid Contents (TFC) Determinations

The quantitative analysis of TPC and TFC in different solvent extracts of *C. montanus* is summarized in Table 3. The ethyl acetate fraction exhibited the highest TPC (47.94 ± 0.32 mg GAE/g), followed by ethanol extract and methanol fraction. Similarly, TFC levels were highest in the ethyl acetate fraction (20.34 ± 1.48 mg rutin/g), followed by ethanol extract and chloroform fraction. The aqueous extract displayed the lowest TPC and TFC.

Table 3. Total phenol content and total flavonoid content of *C. montanus* extracts

Extract	TPC (GAE mg of gallic acid/g)	TFC (mg of rutin /g)
Ethanol*	20.54 ± 0.41	10.27 ± 0.74
Water*	1.03 ± 0.56	0.12 ± 0.06
Petroleum ether**	1.47 ± 0.34	1.63 ± 0.71
Chloroform**	6.55 ± 0.50	9.71 ± 2.03
Ethyl acetate**	47.94 ± 0.32	20.34 ± 1.48
Methanol**	19.19 ± 0.27	5.28 ± 0.34

Data are shown as the average of triplicates ± SD, n = 3; *Extract; **Fraction.

3.3. Antioxidant Activity

The results indicated that the ethanol extract exhibited the lowest IC_{50} (322.1 ± 39.9 µg/mL), followed by the chloroform - (350.3 ± 68.1 µg/mL) and the petroleum ether- fractions (488.4 ± 109.9 µg/mL). The ethyl acetate fraction showed an IC_{50} of 843.3 ± 171.7 µg/mL, while the methanol fraction had an IC_{50} of 889.9 ± 45.3 µg/mL. The aqueous extract demonstrated the lowest antioxidant activity, with an IC_{50} of 1077.7 ± 60.9 µg/mL.

3.4. Antiproliferative Activity

The cytotoxic effects of the extracts and fractions were evaluated against A549 and H1299 cancer cell lines, as well as against gingival fibroblasts. The chloroform and petroleum ether fractions were excluded from further studies due to their toxicity to normal fibroblast cell lines. The aqueous extract exhibited weak cytotoxicity against the H1299 cell line, with an IC_{50} of 984.4 µg/mL, but showed no activity against the A549 cell line (Table 4 and Figure 1). In contrast, methanol fraction displayed no cytotoxicity against the A549 or H1299 cancer cell. Ethanol extract and ethyl acetate fraction exhibited strong cytotoxic activity against the A549 and H1299 cell lines without affecting gingival fibroblasts. The IC_{50} for the ethanol extract was 476.5 and 641.2 µg/mL against the A549 and H1299 cell lines, respectively. Similarly, the IC_{50} for the ethyl acetate fraction was 468.0 and 877.6 µg/mL against the A549 and H1299 cell lines, respectively.

Table 4. The IC_{50} (µg/mL) for different extracts of *C. montanus* tested on three cell lines (A549, H1299, and Fibroblasts).

C. montanus extracts	A549 cancer cell (µg/mL)	H1299 cancer cell (µg/mL)	Fibroblast (µg/mL)
Ethyl acetate	468.035	877.55	Nontoxic
Ethanol	476.545	641.175	Nontoxic
Methanol	Nontoxic	Nontoxic	Nontoxic
Aqueous extract	Nontoxic	984.4	Nontoxic

Data are represented as mean ± SD, n=3.

Figure 1. Antiproliferative effect of *C. montanus* extracts on A) A549; B) H1299; and C) fibroblasts. Data are represented as mean ± SEM, n=3.

3.5. Liquid Chromatography-Mass Spectrometry (LC-MS)

The active ethanol extract and ethyl acetate fraction, were subjected to LC-MS analysis to identify the phytochemical composition to link the identified compounds with their cytotoxic activities. The LC-MS analysis of the ethanol extract revealed the identification of 14 compounds (Table 5).

Table 5. Identified phytochemicals in the ethanol and ethyl acetate extracts of *C. montanus* by LC-MS, expressed as % within identified phytochemicals

Rt	Phytochemicals	Molecular Formula	Ethanol extract	Ethyl-acetate extract
1.9	2,5-dihydroxybenzoic acid	$C_7H_6O_4$	-	10.6
3.1	Chlorogenic acid	$C_{16}H_{18}O_9$	22.5	16.7
3.5	Caffeic acid	$C_9H_8O_4$	3.5	20.6
5.7	Scopoletin	$C_{10}H_8O_4$	1.1	0.1
5.8	Taxifolin	$C_{15}H_{12}O_7$	-	3.9
6.0	Spiraeoside	$C_{21}H_{20}O_{12}$	8.9	8.9
6.4	Aesculetin	$C_9H_7O_4$	1.3	-
6.2	Rosmarinic acid	$C_{18}H_{16}O_8$	1.0	0.1
6.3	5-Glc tricin	$C_{23}H_{24}O_{12}$	-	3.2
6.7	Quercetin	$C_{21}H_{20}O_{11}$	-	7.5
7.0	Myricetin	$C_{15}H_{10}O_8$	0.9	0.1

7.3	Aesculetin	$C_9H_6O_4$	-	0.1
8.9	3,6,2,3-tetrahydroxyflavone	$C_{15}H_{10}O_6$	-	2.4
9.3	Quercetin-4′-O-malonylglucoside	$C_{24}H_{22}O_{15}$	1.5	0.3
9.5	6-methoxyluteolin	$C_{16}H_{12}O_7$	0.2	2.2
10.4	Kaempferol	$C_{15}H_{10}O_6$	1.7	-
10.8	Silymarin	$C_{25}H_{22}O_{10}$	0.5	0.1
11.0	5,6,4-trihydroxy-7.3-dimethoxy flavone	$C_{17}H_{14}O_7$	0.9	0.1
11.1	Sorbifolin	$C_{16}H_{12}O_6$	14.4	-
13.3	Nevadensin	$C_{18}H_{16}O_7$	41.6	23.1

Seventeen compounds were identified in the ethyl acetate fraction using LC-MS. Similar to the ethanol extract, nevadensin and chlorogenic acid were identified as the major compounds in this fraction, along with caffeic acid. The detailed composition is summarized in Table 5.

3.6. Characterization of Ethanol Extract-Loaded Nanoemulsion (NE)

3.6.1. Centrifugation

The stability of the S4 NE was evaluated through centrifugation at 3500 rpm for 15 min. post-centrifugation, all formulations remained homogeneous and transparent, confirming their stability.

3.6.2. Heating/Cooling Cycles

To further assess the stability, S4 NE was subjected to six heating and cooling cycles and three freezing and thawing cycles. No signs of instability, such as cracking, creaming, or separation, were observed throughout these cycles, demonstrating a stable NE.

3.6.3. Morphology and Size

The TEM images of S4 NE revealed that the droplets were uniformly distributed, distinct, and spherical in shape, with no aggregation. The droplet sizes measured by TEM were 12.49 nm and 20.14 nm as shown in Figure 2, which aligned closely with the results obtained using a nanosizer of 13.6 ± 0.5 nm.

Figure 2. TEM image of S4 NE of spherical-shaped droplets.

3.6.4. pH Measurement

The initial pH measurement of S4 NE was 5.39 ± 0.06. After 1 and 2 months of storage at RT (25 ± 2 °C), the pH was 5.36 ± 0.04 and 5.02 ± 0.15. It has been shown that the pH of liquid preparations intended for pulmonary delivery should ideally fall between 3.0 and 8.5, corresponding to the pH of lung fluid (23).

Hence, the pH values were within the acceptable range specified by the European Pharmacopeia for pulmonary medicines, making these formulations suitable for lung delivery(23).

3.6.5. Viscosity Measurement

The viscosity curve of S4 NE demonstrated a shear-thinning behavior, as illustrated in Figure 3. This behavior indicates that the material becomes less viscous as the shear rate increases.

Figure 3. Viscosity curve of S4 NE. Data is presented as mean ± SD (n=3).

3.6.6. Physical Stability

The physical evaluation of S4 NE after two months of storage at RT and 4 °C revealed that the formulation remained transparent and uniform with no color change.

3.6.7. Stability of Droplet Size, Polydispersity Index (PDI), and Zeta Potential (ZP) Measurements

The stability of S4 NE was evaluated by monitoring changes in mean droplet size (MDS) and PDI over a two-month period under two storage conditions: RT and 4 °C. Initial measurements showed that the droplet size and PDI were within the acceptable range for nanoemulsions <100 nm(21).

Over time, both parameters exhibited a gradual increase, but the droplet size remained below 100 nm, indicating good stability. Detailed numerical results for MDS and PDI at each time point are provided in Table 6.

The stability studies confirmed that S4 NE maintained its physicochemical properties, including droplet size, PDI, and zeta potential, within acceptable ranges over two months of storage at both, RT and 4 °C. The gradual increase in droplet size and PDI did not compromise the nanosized nature of the formulation, while the zeta potential measurements indicated sufficient stability despite being below the conventional threshold. These findings highlight the robustness of S4 NE for potential therapeutic applications (24, 25).

Table 6. The stability measurements of MDS, PDI, and ZP of S4 NEs at RT and 4 ºC.

**Time**	**Stored at RT**			**Stored at 4 ºC**		
	**MDS**	**PDI**	**ZP**	**MDS**	**PDI**	**ZP**
**Initial**	13.6±0.5	0.1±0.05	-1.7±0.6	13.6±0.5	0.1±0.05	-1.7±0.6
**1 week**	13.9±0.06	0.2±0.02	-6.7±1.7	13.4±0.2	0.2±0.01	-3.5±1.7
**2 weeks**	16.3±0.1	0.3±0.01	-6.1±0.4	12.6±0.6	0.1±0.01	-6.0±0.4
**3 weeks**	20.5±0.33	0.3±0.005	-6.8±0.4	23.9±0.5	0.3±0.01	-10.2±1.6
**1 month**	25.7±0.73	0.3±0.03	-6.6±3.0	17.8±0.2	0.2±0.04	-12.2±0.04

Data are presented as mean ± SD (n=3).

3.7. Cytotoxicity Evaluation of C. montanus NE

The cytotoxic effects of *C. montanus* NE were evaluated against A549 and H1299 cancer cell lines and normal fibroblasts using the MTT assay (Table 7 and Figure 4). Experiments were performed in triplicate, and results are expressed as mean ± SD. The IC_{50} values for *C. montanus* NE are given in Table 7. A549 and

H1299 cells exhibited similar sensitivity to the NE, while no cytotoxicity was observed against fibroblasts, indicating its selectivity for cancer cells and safety for normal cells.

Table 7. The IC_{50} (µg/mL) for *C. montanus* NE tested on A549, H1299, and fibroblasts cells.

	A549 (µg/mL)	H1299 (µg/mL)	Fibroblast (µg/mL)
***C. montanus* extract**	383.5 ± 7.061	343 ± 6.24	1038.3 ± 62.11

Data are represented as mean ± SD, n=3.

Figure 4. Cytotoxicity profile and IC_{50} of *C. montanus* NE when used to treat **A)** A549; **B)** H1299; and **C)** Fibroblasts cell lines. The X-axis shows the log concentration in µM/mL, and the Y-axis shows the percentage of inhibited cells.

4. Discussion

The phytochemical analysis of *C. montanus* revealed a diverse composition of volatile and non-volatile bioactive compounds. A recent study by Abu-Darwish et al. (2022) identified sesquiterpene hydrocarbons (47.97%) and oxygenated sesquiterpenes (35.32%) as the major volatile compounds in the aroma profile of wild-growing *C. montanus* collected from Jordan (13). Their analysis utilized dried plant material, whereas the present study employed fresh plant material for SPME analysis. These differences may be attributed to environmental factors, such as soil characteristics, temperature variations, and other climatic conditions, in addition to laboratory methodologies. Notably, Abu-Darwish collected plant material from the Mediterranean biogeographic zone of Jordan, while in this study, mature plants were sourced from the Irano-Turanian biogeographic zone (13, 26).

The findings highlight the impact of extraction techniques on the volatile profile of *C. montanus*. Hydro-distillation yielded a broader spectrum of volatile compounds, particularly sesquiterpenes, while SPME selectively enriched monoterpenes, predominantly oxygenated forms. The significant abundance of α-phellandrene (28.60%) and 1,8-cineole (23.30%) suggests their pharmacological relevance to the ethanol extract, given their solubility properties (27, 28).

The TPC and TFC analyses demonstrated the influence of solvent polarity on the extraction efficiency of bioactive compounds. Ethyl acetate, a semi-polar solvent, yielded the highest TPC (47.94 ± 0.32 mg GAE/g) and TFC (20.34 ± 1.48 mg rutin/g), followed by ethanol, which also exhibited considerable extraction efficiency (TPC: 20.54 ± 0.41 mg GAE/g; TFC: 10.27 ± 0.74 mg rutin/g). In contrast, water and non-polar solvents like petroleum ether showed significantly lower extraction capacities. The strong correlation between TPC and TFC suggests that the polyphenolic fraction of *C. montanus* is predominantly composed of flavonoids, which are known for their antioxidant and cytotoxic activities (29, 30). These results, in conjunction with antioxidant activity assessments, underscore the phytochemical richness of *C. montanus* and its potential as a therapeutic botanical resource, particularly when extracted using ethyl acetate and ethanol.

Cytotoxicity evaluations revealed that the ethanol extract and ethyl acetate fraction exhibited promising selective cytotoxic effects on cancer cell lines while sparing normal fibroblasts. Recent studies further support the pharmacological potential of *C. montanus*. Elhady et al. reported that *C. montanus* extract and

its isolated flavonoid, jaceidin, exhibited strong cytotoxic activity against HepG2 and MCF-7 cells, with *in vivo* studies demonstrating tumor-suppressing effects via VEGF inhibition and oxidative stress modulation (8). Additionally, Aidy et al. found that the methanol and petroleum ether extracts of *C. montanus* displayed significant cytotoxicity against colorectal cancer cell lines and promoted colorectal cancer stem cell differentiation through modulation of key genes and microRNA expressions (9).

The ethanol extract's major identified compound, nevadensin (5,7-dihydroxy-6,8,4′-trimethoxyflavone), is known for its diverse biological activities, including hypotensive, anti-inflammatory, antimicrobial, and anticancer properties (31). Studies have identified nevadensin as a selective inhibitor of human carboxylesterase 1 and topoisomerases TOPO I and TOPO IIα using in vitro models (32, 33).

Chlorogenic acid, a widely distributed phenolic acid, exhibits multiple biological effects, including hepatoprotective, antimicrobial, immunomodulatory, antioxidant, antidiabetic, and anticancer properties. Studies have demonstrated its role in cancer prevention through mechanisms such as inhibition of cell division, angiogenesis suppression, cell cycle arrest, and apoptosis induction, supported by in vitro, in vivo, and clinical studies (34, 35). Notably, chlorogenic acid has been approved by the China Food and Drug Administration as a natural anticancer drug (34).

Sorbifolin (Scutellarin-7-methylether), another major compound in the ethanol extract, is recognized for its antioxidant, anticancer, antimicrobial, anti-inflammatory, and antidiabetic activities(36). *In silico* studies by Shah and Kin concluded that sorbifolin is a potent candidate for Ewing sarcoma treatment (37).

These findings suggest that the identified phytochemicals, particularly flavonoids, play a significant role in the cytotoxic activity observed in the ethanol extract. Given the well-established antioxidant properties of phenolics and flavonoids, their ability to neutralize oxygen radicals *via* redox mechanisms may contribute to their antiproliferative effects (38). Further research is warranted to elucidate their specific mechanisms of action.

To enhance pulmonary delivery for lung carcinoma treatment, nanoemulsions (NEs) were developed. The optimized S4 NE formulation demonstrated excellent stability and an appropriate droplet size for pulmonary administration. Cytotoxicity evaluations revealed that *C. montanus* NE selectively targeted cancer cells (A549 and H1299) while sparing normal fibroblasts, supporting its potential as a safe and effective anticancer formulation. These findings emphasize the therapeutic promise of *C. montanus* NE for targeted cancer therapy, warranting further investigation into its mechanisms of action and *in vivo* efficacy.

5. Conclusions

This comprehensive study on *C. montanus* highlights its significant phytochemical diversity and pharmacological potential. The GC-MS analysis revealed that HD hydro-distillation, produces a more diverse composition rich in sesquiterpenes, while SPME was selectively enriched with monoterpenes. The quantitative analysis of TPC and TFC demonstrated that ethyl acetate fraction and ethanol extract contain large amounts of these bioactive compounds, aligning with their strong antioxidant and cytotoxic activities. Ethanol extract and ethyl acetate fraction showed promising selective cytotoxicity against A549 and H1299 cancer cell lines, without affecting normal fibroblasts, underlining their potential for therapeutic applications.

LC-MS analysis identified key phytochemicals, including nevadensin and chlorogenic acid, which likely contribute to the observed bioactivities. The formulation of an ethanol extract-loaded NEs further enhanced

the delivery potential of *C. montanus* extracts, with S4 NE demonstrating excellent stability, suitable droplet size, and pH for pulmonary delivery. The cytotoxicity evaluation of the NE confirmed its selective anticancer activity, with no adverse effects on normal cells.

Overall, this study underscores the therapeutic potential of *C. montanus* as a rich source of bioactive compounds with antioxidant, cytotoxic, and anticancer properties. The findings suggest that *C. montanus* extracts, particularly when formulated as NEs, are promising natural therapeutic agents, especially in cancer treatment. Future studies should focus on elucidating the mechanisms of action of the identified compounds and exploring their *in vivo* efficacy and safety profiles.

Author Contributions: Conceptualization, N.H. and F.A.; methodology, N.H., H.A, H.S. and I.A; software, N.H., M.W. and E.A.; validation, N.H., R.H. and M.W.; formal analysis, N.H.; investigation, N.H. and F.A.; resources, N.H.; data curation, N.H.; writing—original draft preparation, H.A. and H.S.; writing—review and editing, N.H., R.H. M.W. and F.A.; visualization, N.H.; supervision, N.H. and F.A.; project administration, N.H.; funding acquisition, N.H.; Plant material collection and identification, F.A and I.A. All authors have read and agreed to the published version of the manuscript.

Funding: This research was funded by Deanship of Scientific Research and Innovation at Al-Zaytoonah University of Jordan (2022-2023/07/23).

Data Availability Statement: All data generated or analyzed during this study are included in this published article.

Conflicts of Interest: The authors declare no conflicts of interest. The funders had no role in the design of the study; in the collection, analyses, or interpretation of data; in the writing of the manuscript; or in the decision to publish the results.

Abbreviations

The following abbreviations are used in this manuscript:

DLS	Dynamic Light Scattering
GC-MS	Gas Chromatography-Mass Spectrometry
HD	HydroDistillation
LC-MS	Liquid Chromatography-Mass Spectrometry
MDS	Mean Droplet Size
NEs	Nanoemulsions
PDI	PolyDispersity Index
SPME	Solid-Phase Micro-Extraction
TEM	Transmission Electron Microscopy
TFC	Total Flavonoid Content
TPC	Total Phenol Content
ZP	Zeta Potential

References

1. Hegazy MF, Matsuda H, Nakamura S, Hussein TA, Yoshikawa M, Paré PW. Chemical constituents and their antibacterial and antifungal activity from the Egyptian herbal medicine Chiliadenus montanus. Phytochemistry. 2014;103:154-61.
2. Castillo LV, García FZ, Arnáez CZ. The Genus Chiliadenus: A Comprehensive Review of Taxonomic Aspects, Traditional Uses, Phytochemistry and Pharmacological Activities. Plants. 2025;14(2):205.
3. Afifi FÜ, Al-Khalil S, Abdul-Haq BK, Mahasneh A, Al-Eisawi DM, Sharaf M, et al. Antifungal flavonoids from Varthemia iphionoides. Phytotherapy Research. 1991;5(4):173-5.
4. Afifi F, Aburjai T. Antiplatelet activity of Varthemia iphionoides. Fitoterapia. 2005;75:629-33.
5. Al-Dabbas M, Fumio H, Abdelgaleil S, Suganuma T, Akiyama K, Hayashi H. Antibacterial activity of an eudesmane sesquiterpene isolated from common Varthemia, Varthemia iphionoides. Journal of Ethnopharmacology. 2005;97:237-40.
6. Soliman FM, Moussa MY, Abdallah H, Othman SM. Cytotoxic activity of flavonoids of Jasonia Montana Vahl. (Botsch). (Astraceae) growing in Egypt. Australian Journal of Basic and Applied Sciences, 2009;3(1):148-52.
7. Hussein MA, Abdel-Gawad SM. Protective effect of Jasonia montana against ethinylestradiol-induced cholestasis in rats. Saudi pharmaceutical journal : SPJ : the official publication of the Saudi Pharmaceutical Society. 2010;18(1):27-33.
8. Elhady SS, Eltamany EE, Shaaban AE, Bagalagel AA, Muhammad YA, El-Sayed NM, et al. Jaceidin Flavonoid Isolated from Chiliadenus montanus Attenuates Tumor Progression in Mice *via* VEGF Inhibition: In Vivo and In Silico Studies. Plants. 2020;9(8).
9. Aidy EA, Fathy Z, Ahmed WA, El-Gendy S, Abou-Bakr A, Omran M, et al. Jasonia montana Extracts Stimulate Cell Differentiation in HCT-116 and Caco-2 Cell Lines %J Egyptian Journal of Chemistry. 2022;65(12):165-74.
10. El-Bsoumy E, Habib E, Ibrahim A, ahmed s. Phytochemical constituents and biological activities of Jasonia montana (Asteraceae). Records of Pharmaceutical and Biomedical Sciences. 2021;5:1-11.
11. Abu-Darwish D, Shibli R, Al-Abdallat AM. Phenolic Compounds and Antioxidant Activity of Chiliadenus montanus (Vhal.) Brullo. grown in vitro. Jordan Journal of Pharmaceutical Sciences. 2024;17(3):611-28.
12. shaaban a, El Tamany E, Ayyad S, ahmed s. Genus chiliadenus is a unique supplier of several chemical flocks: A review. Records of Pharmaceutical and Biomedical Sciences. 2021;5:20-40.
13. Abu-Darwish D, Shibli R, Al-Abdallat AM. In Vitro Cultures and Volatile Organic Compound Production in Chiliadenus montanus (Vhal.) Brullo. Plants. 2022;11(10).
14. Al-Eisawi. Flora of Jordan Checklist Revised Edition 1. University of Jordan: The University of Jordan Press. Amman, Jordan; 2013.
15. Ferhat MA, Meklati BY, Smadja J, Chemat F. An improved microwave Clevenger apparatus for distillation of essential oils from orange peel. Journal of Chromatography A. 2006;1112(1-2):121-6.
16. Afifi FU, Kasabri, V., & Abaza, I. M. <GC-MS composition and antiproliferative activity of Inula graveolens (L.).pdf>. Arabian Journal of Medicinal and Aromatic Plants. 2015;1:57-66.
17. Adams R. Identification of Essential Oil Components by Gas Chromatography/Quadrupole Mass Spectroscopy. Carol Stream. 2005;16:65-120.
18. Aboalhaija NH, Syaj H, Afifi F, Sunoqrot S, Al-Shalabi E, Talib W. Chemical Evaluation, *In Vitro* and *In Vivo* Anticancer Activity of Lavandula angustifolia Grown in Jordan. Molecules 2022;27(18).

19. Aboalhaija A, Amro R, Abaza I, Khalil E, Al-Aboudi A, Abu Zarga M, et al. Schinus molle L. Collected from Jordan and Turkey: Essential Oil Composition and Anticholinesterase Activity. Journal of Essential Oil Bearing Plants. 2019;22:704-16.
20. Mohammad AA-M, Nour HA, Ismail FA, Mohammad HK, Malek AZ, Fatma UA. Chromatographic Analysis (LC-MS and GC-MS), Antioxidant Activity, Total Phenol and Total Flavonoid Determination of Ononis natrix L. Grown in Jordan. Jordan Journal of Chemistry (JJC). 2021;16(1):31-9.
21. Hamed R, Basil M, AlBaraghthi T, Sunoqrot S, Tarawneh O. Nanoemulsion-based gel formulation of diclofenac diethylamine: design, optimization, rheological behavior and in vitro diffusion studies. Pharmaceutical Development and Technology. 2016;21(8):980-9.
22. Ahmadi O, Jafarizadeh-Malmiri H. Intensification process in thyme essential oil nanoemulsion preparation based on subcritical water as green solvent and six different emulsifiers. Green Processing and Synthesis 2021; 10(1):430-9.
23. Weber S, Zimmer A, Pardeike J. Solid Lipid Nanoparticles (SLN) and Nanostructured Lipid Carriers (NLC) for pulmonary application: a review of the state of the art. European Journal of Pharmaceutics and Biopharmaceutics 2014;86(1):7-22.
24. Hamed R, Abu Alata Wa, Abu-Sini M, Abulebdah DH, Hammad AM, Aburayya R. Development and Comparative Evaluation of Ciprofloxacin Nanoemulsion-Loaded Bigels Prepared Using Different Ratios of Oleogel to Hydrogels. Gels, 2023;9(7):592.
25. Xu XY, Yu Z, Zhu Y, Wang B. Effect of sodium oleate adsorption on the colloidal stability and zeta potential of detonation synthesized diamond particles in aqueous solutions. Diamond and Related Materials - DIAM RELAT MATER. 2005;14.
26. C. P. Biogeography. In M. Ababsa (Ed.), Atlas of Jordan. In: l'Ifpo Pd, editor.
Atlas of Jordan. Beyrout, Lebanon Publi-cation sur OpenEdition Books; 2013. p. 77-87.
27. Shiekh RAE, Atwa AM, Elgindy AM, Mustafa AM, Senna MM, Alkabbani MA, et al. Therapeutic applications of eucalyptus essential oils. Inflammopharmacology. 2025;33(1):163-82.
28. de Cássia da Silveira e Sá R, Andrade LN, de Sousa DP. A review on anti-inflammatory activity of monoterpenes. Molecules. 2013;18(1):1227-54.
29. Ahmed SI, Hayat MQ, Tahir M, Mansoor Q, Ismail M, Keck K, et al. Pharmacologically active flavonoids from the anticancer, antioxidant and antimicrobial extracts of Cassia angustifolia Vahl. BMC Complementary and Alternative Medicine. 2016;16(1):460.
30. Kumar S, Pandey AK. Chemistry and biological activities of flavonoids: an overview. The Scientific World Journal. 2013;2013:162750.
31. Brahmachari G. Nevadensin: Isolation, chemistry and bioactivity. International Journal of Green Pharmacy. 2010;4(4).
32. Wang YQ, Weng ZM, Dou TY, Hou J, Wang DD, Ding LL, et al. Nevadensin is a naturally occurring selective inhibitor of human carboxylesterase 1. International Journal of Biological Macromolecules. 2018;120(Pt B):1944-54.
33. Müller L, Schütte LRF, Bücksteeg D, Alfke J, Uebel T, Esselen M. Topoisomerase poisoning by the flavonoid nevadensin triggers DNA damage and apoptosis in human colon carcinoma HT29 cells. Archives of Toxicology. 2021;95(12):3787-802.
34. Gupta A, Atanasov AG, Li Y, Kumar N, Bishayee A. Chlorogenic acid for cancer prevention and therapy: Current status on efficacy and mechanisms of action. Pharmacological Research. 2022;186:106505.
35. Singh S, Varshney M. Exploring the Pharmacological Potential of Chlorogenic acid as an Anti-Cancer Agent and a Call for Advance Research. Combinatorial Chemistry & High Throughput Screening. 2025; 28(12).
36. Belmehdi O, Mssillou I, Khalid A, Abdalla AN, Almalki M, Alqurashi RS, et al. Unveiling the Biological Activities, Pharmacological Potential, and Health Benefits of Sorbifolin: A Comprehensive Review. Chemistry & Biodiversity 2025;22(2):e202401463.

37. Shah FH, Kim SJ. Inhibitors of EYA3 Protein in Ewing Sarcoma. Asian Pacific Journal of Cancer Prevention : APJCP. 2022;23(5):1539-45.
38. Althaher AR, Shehabi RF, Ameen HH, Awadallah MW, Mastinu A. Calamintha incana Methanolic Extract: Investigation of Phytochemical Composition and Antioxidant and Antibacterial Activities. Journal of Food Biochemistry 2024;2024(1):6634969.

Click here to access/download;Figure;FIG 1A.tif

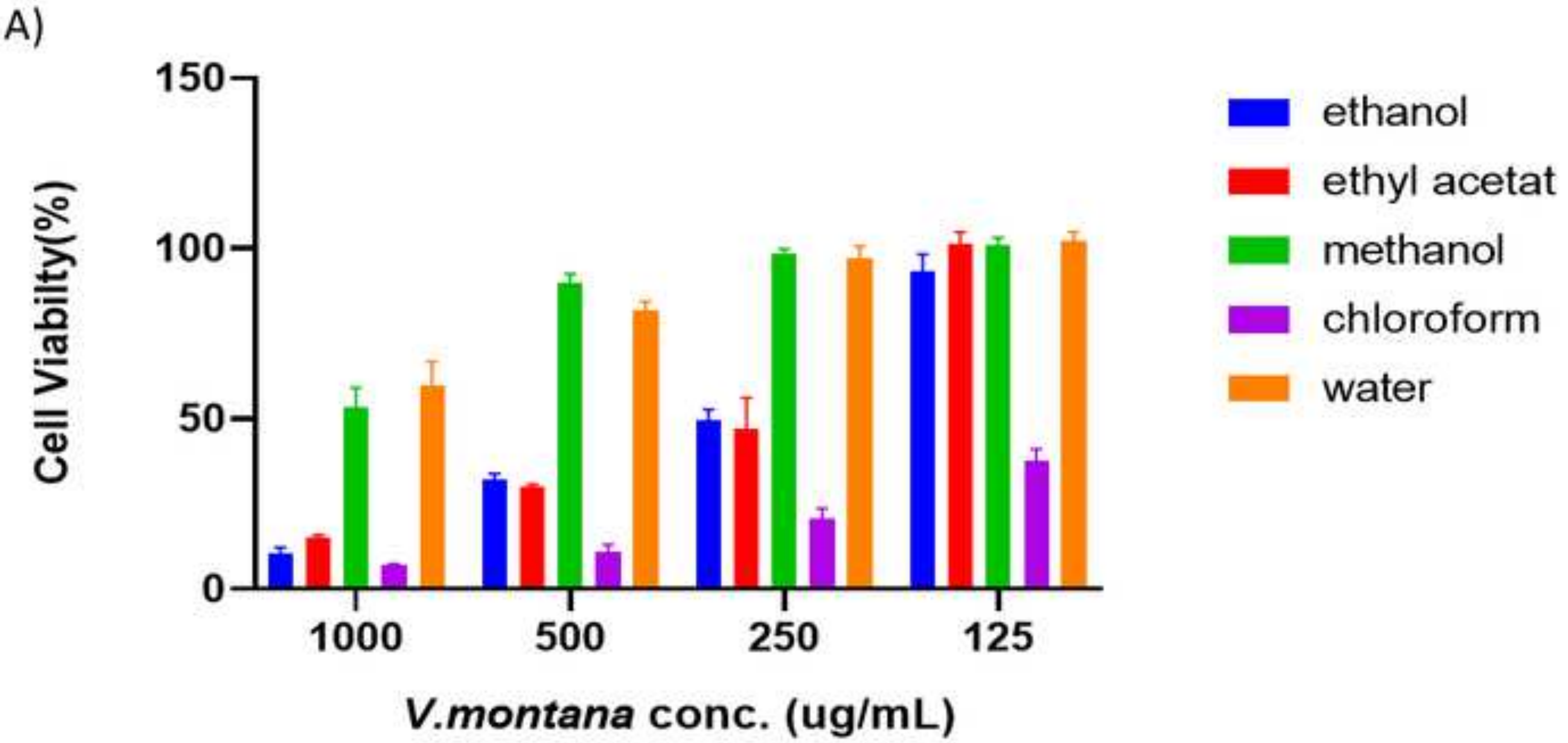

Figure 1B

Click here to access/download;Figure;FIG 1B.tif

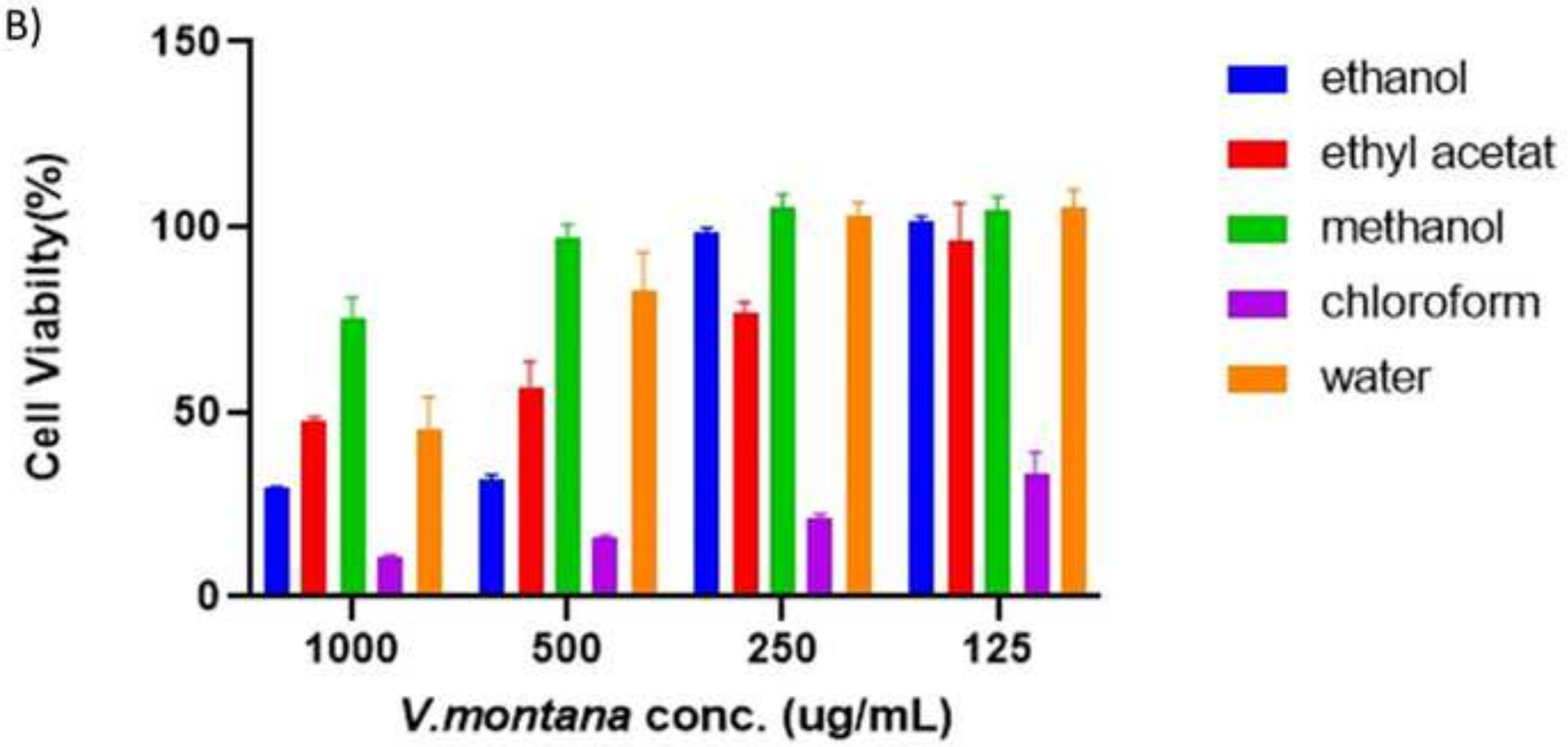

Figure 1C

Click here to access/download;Figure;FIG 1C.tif

C)

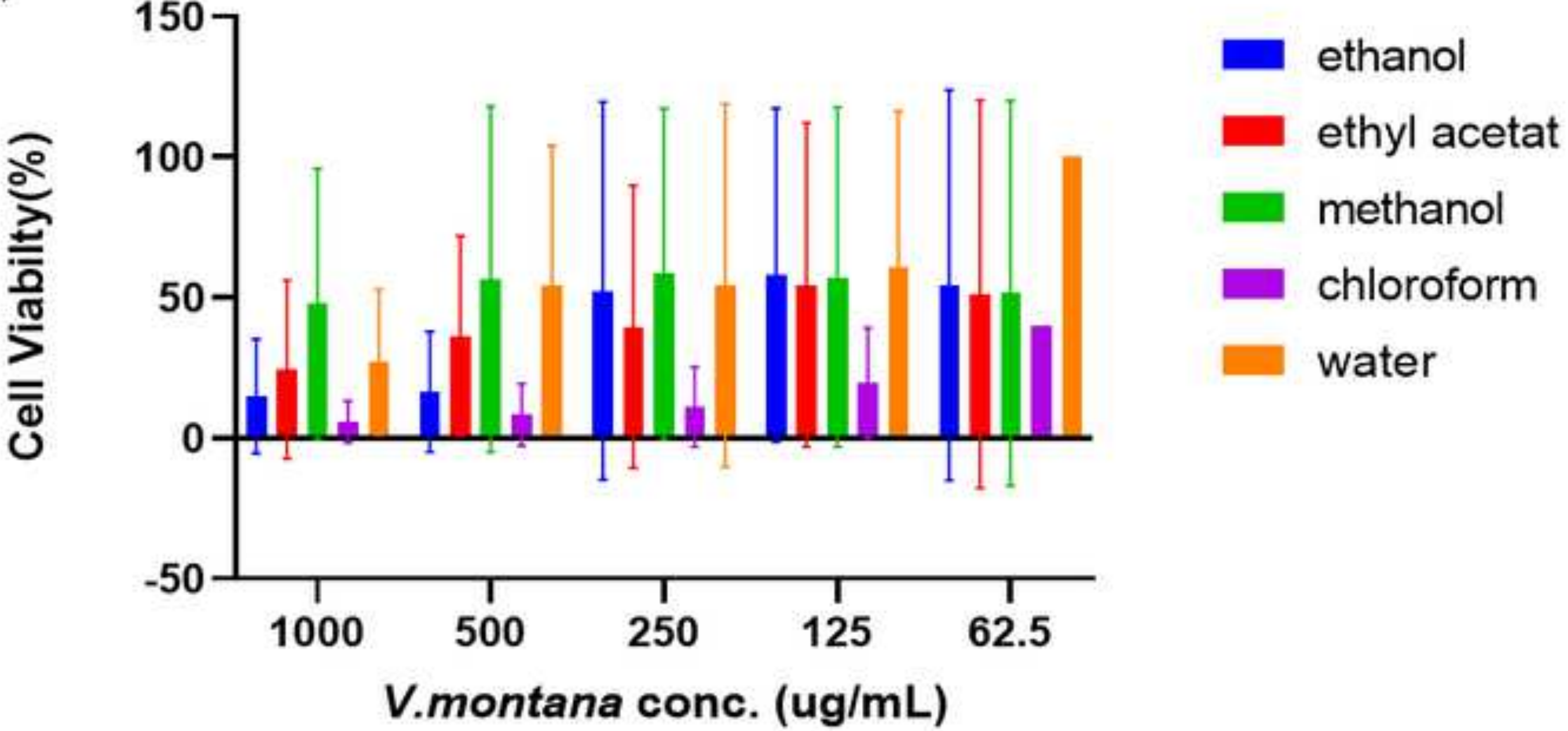

Figure 2

Click here to access/download;Figure;Fig 2.tif

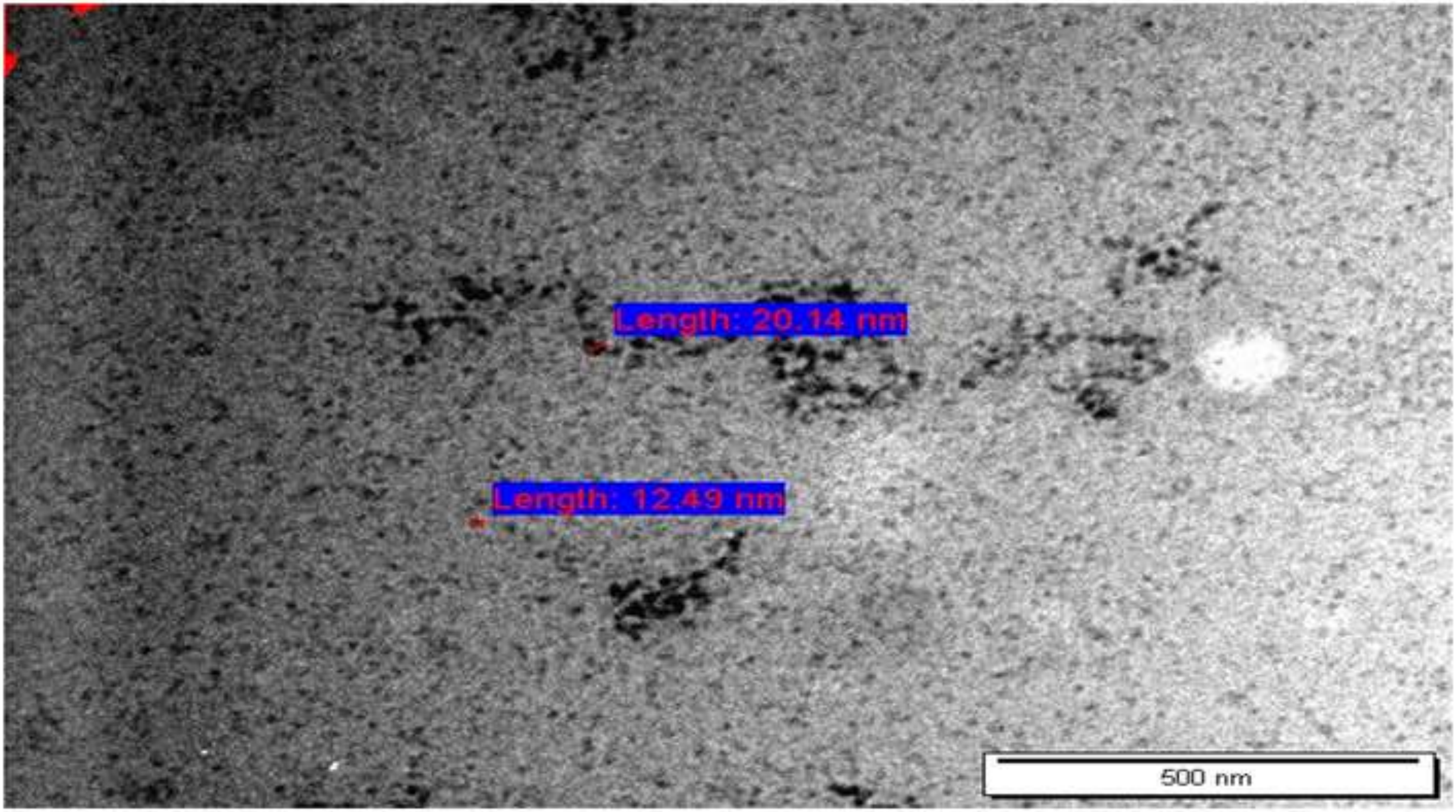

Figure 3

Click here to access/download;Figure;Fig 3.tif

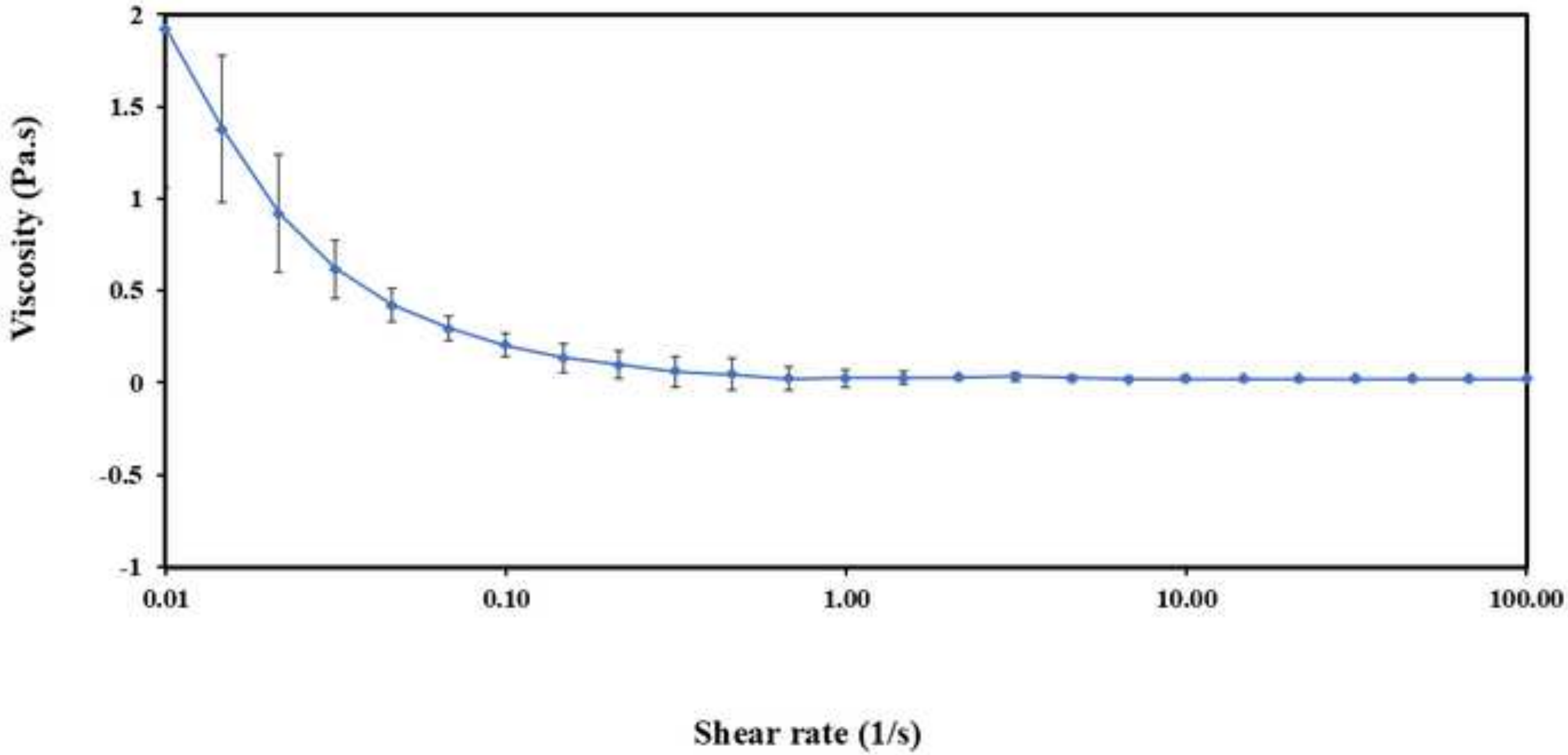

Figure 4A

Click here to access/download;Figure;FIG 4A.tif

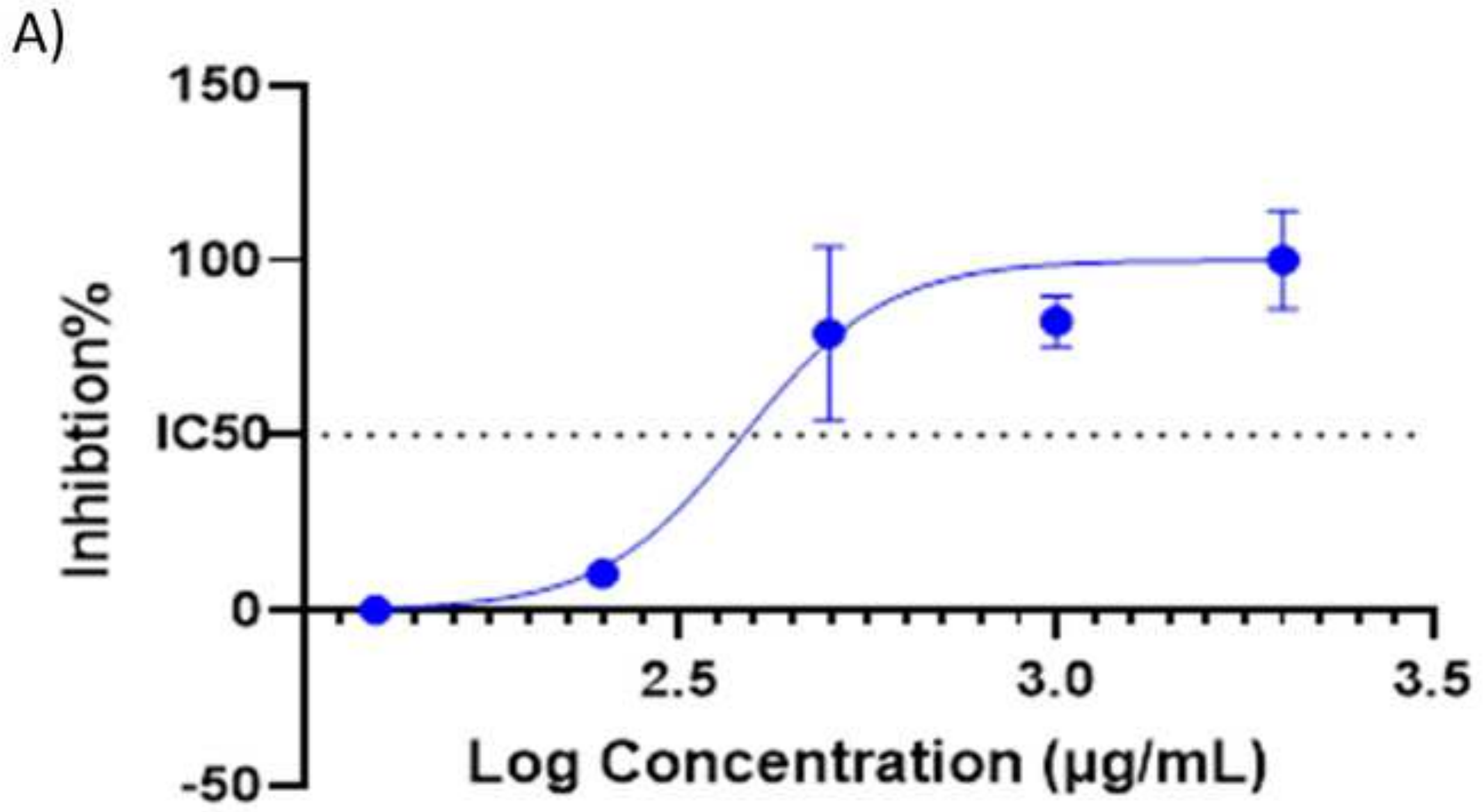

Click here to access/download;Figure;FIG 4B.tif

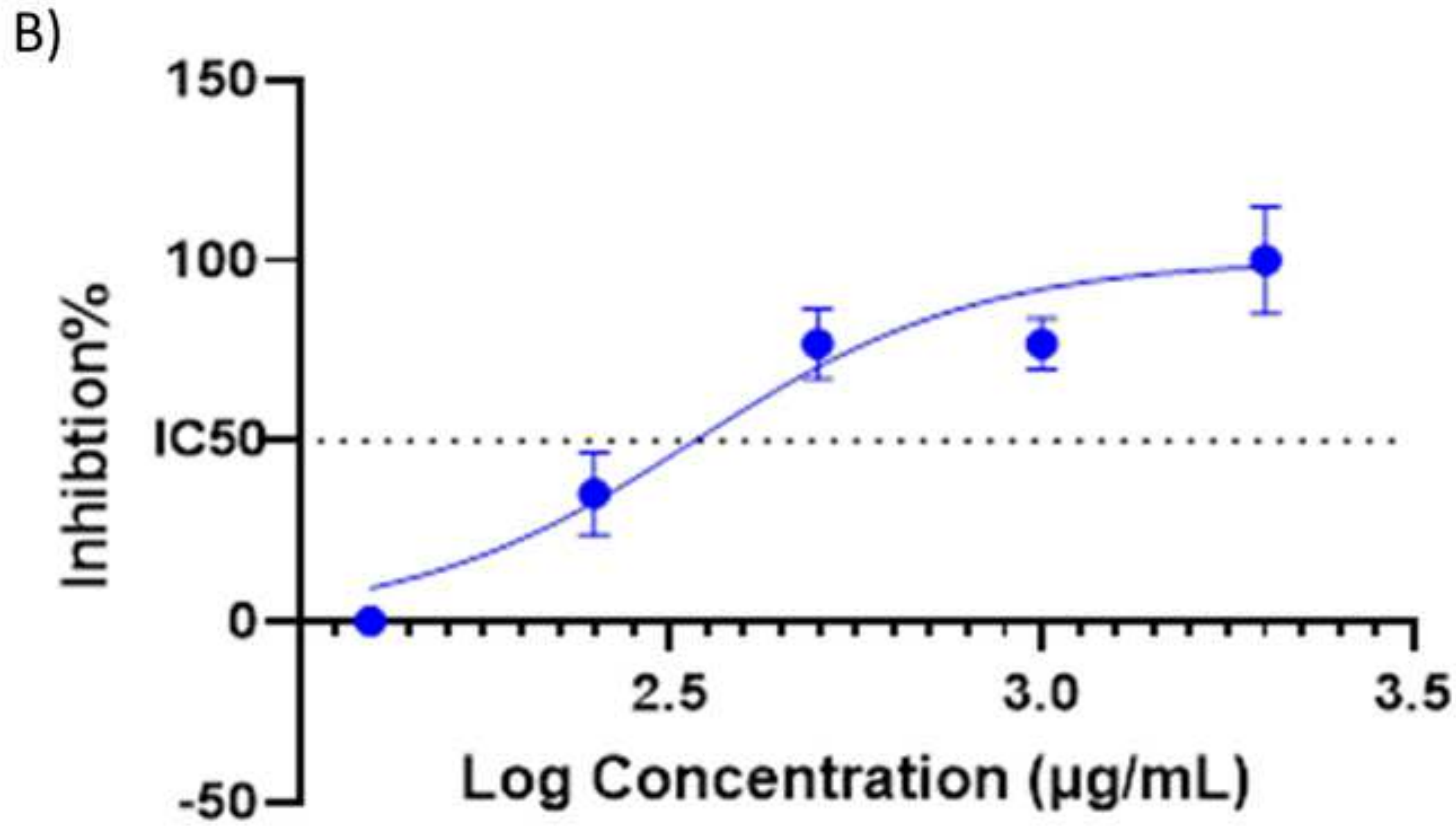

 Click here to access/download;Figure;FIG 4C.tif

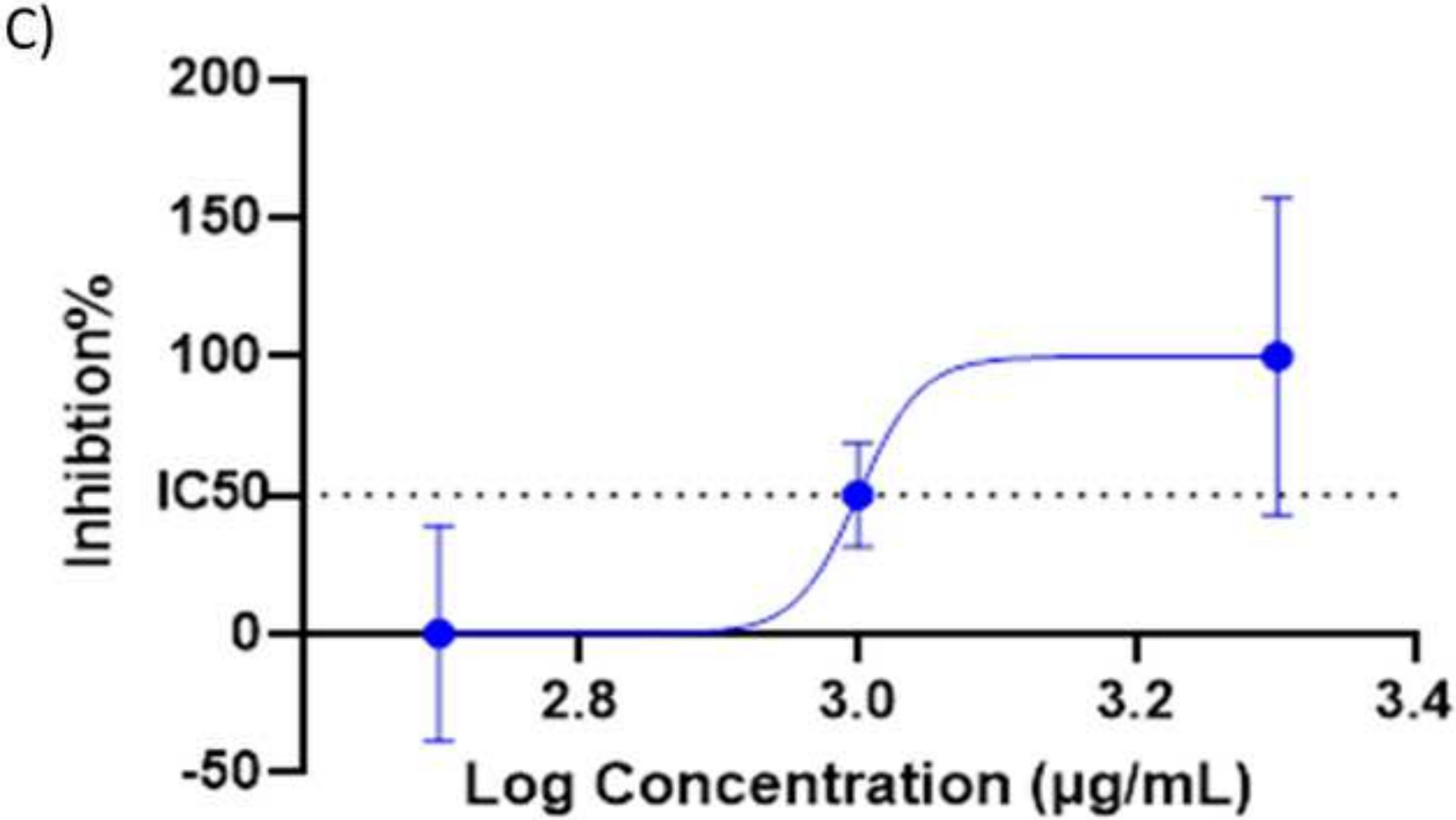

Phytochemical Characterization, Anticancer Potential, and Nanoemulsion-Based Delivery of *Chiliadenus montanus* ~~*Varthemia montana*~~

Nour Aboalhaija [1,*], Hala Abulawi [1], Rania Hamed [1], Mohammad Alwahsh [1], Fatma Afifi [2], Heba Syaj [1], Elham Abusharieh [1] and Ismail Abaza [2]

[1] Department of Pharmaceutical Sciences, Faculty of Pharmacy, Al-Zaytoonah University of Jordan,
Amman 11733, Jordan
[2] Department of Pharmaceutical Sciences, School of Pharmacy, The University of Jordan, 11942 Amman, Jordan
* Correspondence: n.aboalhaija@zuj.edu.jo

Abstract: *Chiliadenus montanus* ~~*Varthemia montana*~~ (Vahl) Boiss. (Asteraceae) is a pharmacologically significant plant with different potent pharmacological properties.~~potential applications in pulmonary drug delivery.~~ This study aimed to evaluate its phytochemical and biological properties and develop nanoemulsions (NEs) to enhance pulmonary delivery for lung carcinoma treatment. Ethanol and water extracts, along with petroleum ether, chloroform, ethyl acetate, and methanol fractions, were assessed for total phenol~~ic~~ and flavonoid contents, antioxidant activity, and cytotoxicity against H1299 and A549 lung cancer cell lines. Ethyl acetate fraction exhibited the highest phenol (47.94 ± 0.32 mg GAE/g) and flavonoid (20.34 ± 1.48 mg rutin/g) contents, while the ethanol extract showed the strongest antioxidant activity (IC_{50} = 322.1 μg/mL) and selective cytotoxicity (IC_{50} = 641.2 μg/mL) against H1299 cells. Nevadensin, chlorogenic acid, and sorbifolin were identified as the major constituents of the ethanol extract using liquid chromatography-mass spectrometry (LC-MS) analysis. Gas chromatography-mass spectrometry (GC-MS) analysis revealed α-phellandrene, 1,8-cineole, and α-cadinol as the major volatile constituents. The major volatile compound of the aroma profile of the aerial parts, determined by solid phase micro extraction (SPME) was 1,8-cineole. Spontaneous emulsification was used to formulate ethanolic NEs, with S3, S4, S5, and S6 demonstrating thermodynamic stability, suitable pulmonary pH, and droplet sizes below 200 nm. These findings highlight the promising potential of *C. montanus* ~~*V*~~. ~~*montana*~~ NEs as stable pulmonary drug delivery systems for lung carcinoma therapy.

Keywords: *Chiliadenus montanus* ~~*Varthemia montana*~~; anticancer; nanoemulsion; pulmonary delivery; LC-MS; SPME; GC-MS

1. Introduction

~~The genus *Varthemia* (Asteraceae, formerly Compositae) comprises over 91 genera and includes three species native in Jordan: *Varthemia montana* Vahl (Boiss.), *V. candicans* Delile (Boiss.), and *V. iphionoides* Boiss. & Blanche (1). Among these, *V. montana* (Vahl) Boiss (syn. *Chiliadenus montanus* (Vahl.) Brullo; *Jasonia montana* (Vahl.) Botsch., *Chrysocoma montana* (Vahl.) Symb., *Inula conyzoides* DC., *Linosyris montana* DC., *Varthemia conyzoides* Boiss.) commonly known as desert *Varthemia*, is a perennial bushy herb characterized by small leaves, numerous branches, and aromatic, hairy, and sticky stems, reaching a height of 20-50 cm (2). It thrives in rocky and semi-arid regions, blooming between September and December with distinctive tubular yellow flowers(2).Various *Varthemia* species have demonstrated significant pharmacological~~

~~activities including antibacterial, antifungal, antioxidant, anti-inflammatory, anticancer, antispasmodic, and antiplatelet effects (2-5)~~

The genus *Chiliadenus* (Asteraceae, formerly Compositae) includes three species native in Jordan: *C. montanus* (Vahl) Brullo (syn. *Varthemia montana* Vahl (Boiss.)), *C. candicans* (Delile) Brullo (syn. *V. candicans* Delile (Boiss.)), and *C. iphionoides* (Boiss. & Blanche) Brullo (syn. *V. iphionoides* Boiss. & Blanche) (1). Among these, *C. montanus*, commonly known as desert Varthemia, is a perennial bushy herb characterized by small leaves, numerous branches, and aromatic, hairy, and sticky stems, reaching a height of 20–50 cm (2). It thrives in rocky and semi-arid regions, blooming between September and December with distinctive tubular yellow flowers (2). Various species formerly assigned to *Varthemia* have demonstrated significant pharmacological activities, including antibacterial, antifungal, antioxidant, anti-inflammatory, anticancer, antispasmodic, and antiplatelet effects (2-5).

Phytochemically, *C. montanus* ~~*V. montana*~~ is known to be rich in volatile oils, in volatile and non-volatile terpenoids and in flavonoids, -mainly methoxylated-. All these secondary metabolites are associated with a wide range of biological activities, including antibacterial, antifungal, antioxidant, anti-inflammatory, anticancer, antispasmodic, antiplatelet and hypoglycemic effects (2, 6-12)
The species' possible benefits in treatment of Alzheimer's disease are suggested by El-Bsoumy et al. (10). Several researchers have also emphasized the different traditional uses of *C. montanus* ~~*V. montana*~~ including its benefits in management of diarrhea, kidney problems, stomachaches, and chest ailments, especially recommended in Mediterranean countries (2, 12, 13). Keeping in mind that environmental factors can significantly influence the phytochemical profiles of medicinal plants, further investigations of their extracts is warranted to better understand its traditional uses.
This present study aimed to evaluate~~,~~ the chemical composition and biological activities of extracts and fractions of *C. montanus* ~~*V. montana*~~ growing in Jordan. Their total phenol and flavonoid contents ~~TPC and TFC~~ were analyzed, along with their antioxidant and antiproliferative activities. To enhance and expand its phytochemical and biological activities, pharmaceutical formulation of its ethanol extract was approached. The ethanol extract of *C. montanus* ~~*V. montana*~~ was incorporated into a NE formulation and assessed as a potential drug delivery system for lung cancer therapy. By investigating its cytotoxic potential and suitability for pulmonary delivery, this research highlights the therapeutic value of *C. montanus*~~*V. montana*~~ and its potential role as adjunctive phytotherapy for conventional chemotherapy. The composition of the active extract was evaluated by LC-MS. Additionally, volatile oil composition of the hydro-distilled (HD) essential oil and aroma profile of the aerial parts of this species were determined to obtain a complete picture of its phytochemical composition.

2. Materials and Methods

2.1. Plant Material

Chiliadenus montanus ~~*Varthemia montana*~~ was collected from Balqa region (32.037° N, 35.7288° E) of Jordan, in late September 2022. The plant was authenticated by one of the authors using descriptive references and in comparison, with the herbarium specimen in the School of Pharmacy, The University of Jordan, Amman. Voucher specimen were kept in the School of Pharmacy (FMJ-AST95-3) *C. montanus* ~~*V. montana*~~ is characterized by its multiple branches, hairy appearance, sticky stems, and bushy perennial form with small leaves and a woody base, typically ranging in length from 20 to 50 cm (14).

2.2. Essential Oil Extraction by hydro-distillation and Aroma Profile Determination by Solid-Phase Micro-Extraction (SPME)

For the isolation of the essential oil of *C. montanus* ~~*V. montana*~~ aerial parts of the plant were subjected to hydro-distillation in a Clevenger apparatus for 3 hours. The resulting oil was pooled, dried over anhydrous sodium sulfate (Na_2SO_4) and stored at 4 ºC in amber glass vials until analysis (15). For aroma profiling, solid-phase micro-extraction (SPME) was employed to capture and analyze the volatile compounds as described earlier (16).

2.3. Gas Chromatography-Mass Spectrometry (GC-MS)

The volatile organic compounds emitted from *C. montanus* ~~*V. montana*~~ essential oil were analyzed using GC-MS (16). Compound identification was performed using internal digital libraries, including those from NIST and Wiley, by comparing the acquired retention indices (RI) with published values for C_8-C_{20} *n*-alkanes, measured under similar conditions (17).

The experimental RI values were calculated using the following formula:

RI=100 (n + (N - n) x (Log tr (unknown) – logtr (n)) / logtr(N) – logtr(n)

where RI represents the retention time measured from the time of the unretained small molecule, and *n* and *N* represent the numbers of carbon atoms in the smaller and larger alkanes, respectively. A Hewlett-Packard HP-8590 gas chromatograph equipped with a split-spitless injector (split ratio 1:50) and a flame ionization detector (FID) were utilized for quantitative analysis (% area). The concentration of the identified compounds was determined by measuring the relative peak areas of the oil components. ~~compared to published values for C_8 -$C2_0$ *n*-alkanes.~~

2.5. Extracts Preparation

In this study, the ethanol extract of *C. montanus* ~~*V. montana*~~ was prepared by soaking the dried and crushed aerial parts in 70% ethanol for three weeks at room temperature (RT). Following this period, ethanol was evaporated under vacuum using a rotary evaporator set at 40 °C. The crude extract was first subjected to defatting by liquid-liquid extraction using petroleum ether in a separatory funnel to remove chlorophyll and fatty materials. This process was repeated until the petroleum ether layer became colorless, ensuring the complete removal of non-polar impurities. The defatted aqueous methanol layers were collected and subsequently fractionated based on polarity using a sequential liquid-liquid extraction approach. Chloroform was employed to isolate non-polar constituents, followed by ethyl acetate to extract intermediately polar phytochemicals. The remaining aqueous methanol layer was designated as the methanol fraction, containing the highly polar components. Additionally, aqueous and ethanol extracts were prepared by soaking the plant material in water and 70% ethanol, respectively, without further fractionation.

2.6. Determination of Total Phenol (TPC) and Total Flavonoid Contents (TFC)

The TPC of *C. montanus* ~~*V. montana*~~ extracts was determined using the Folin-Ciocalteu method. Gallic acid in methanol served as a standard, and five serial dilutions (100, 50, 25, 12.5, and 6.125 µg/mL) were

prepared. Absorbance readings were taken at 765 nm in triplicate, with methanol used as a blank. The total phenol content was expressed as gallic acid equivalents (GAE) (18).

To determine TFC, aluminum chloride was utilized. After incubation for 30 min at RT, the absorbance was measured at 415 nm. Rutin served as the calibration standard, with five concentrations (100, 50, 25, 12.5, and 6.125 μg/mL) prepared in methanol. Experiments were carried out in triplicate, and the results were averaged (18).

2.7. Antioxidant Activity

To prepare the DPPH reagent, approximately 4 mg of DPPH powder was dissolved in 100 mL of methanol in an Erlenmeyer flask. The mixture was agitated using a magnetic stirrer at 60 to 100 rpm for 5 min to ensure complete dissolution. To protect the DPPH solution from light and evaporation, the flask was wrapped in foil and sealed. Ascorbic acid was used as a standard to establish a calibration curve. Ten different concentrations were prepared (1000, 500, 250, 125, 62.5, 31.25, 15.625, 7.815, 3.91, and 1.955 μg/mL). In a test tube, 3 mL of the DPPH solution was mixed with 2 mL of each extract. The reaction was allowed to proceed for 30 min at RT in complete darkness.

After the incubation period, the absorbance of each extract was measured at 517 nm. The percentage of inhibition was calculated using the following formula:

$$I\ \% = (A^{\circ} - A\varkappa) / A^{\circ} \times 100$$

where A° represents the absorbance of the control (DPPH solution without extract), and $A\varkappa$ is the absorbance of the extract at a specific concentration. The IC_{50} value was determined as the concentration required to inhibit 50% of the DPPH radicals.

2.8. In Vitro Cytotoxicity on Cancer Cell Lines

Human cancer cell lines A549, H1299, and normal fibroblasts were obtained from the American Type Culture Collection (ATCC, Manassas, VA, USA). A549 (CCL-185) and H1299 (CRL-5803) cells were cultured in RPMI-1640 medium, while fibroblasts (PCS-201-018) were cultured in Dulbecco's Modified Eagle's Medium (DMEM). Both media were supplemented with 10% fetal bovine serum (Cytevia, Austria), 1% L-glutamine, penicillin-streptomycin, and HEPES buffer (EuroClone S.P.A., Italy). Cells were incubated at 37 °C in a humidified 5% CO_2 atmosphere and sub-cultured every 2–3 days, with passages limited to 20 (19) Cells were seeded into 96-well plates (10,000 cells/100 μL/well) (SPL Life Sciences Co., Korea). After reaching 70% confluence, cells were treated with serially diluted *C. montanus* ~~*V. montana*~~ extract (2000, 1000, 500, 250, and 125 μM) in five replicates. After 48 hours of incubation, media were replaced with fresh media containing 15 μL of MTT (3-[4,5-dimethyl-thiazol-2-yl]-2,5-diphenyl tetrazolium bromide; ATCC® 30-1010 K) and incubated for 3 hours. Formazan crystals were dissolved with solubilization solution, and absorbance was measured at 570 nm using an HTX multi-mode microplate reader (BioTek, USA). Data were analyzed using GraphPad Prism 8.0.2.

2.9. LC-MS Analysis

Extracts were dissolved in solvent and chromatographic separation was achieved using Burker Daltonik (Bremen, Germany) impact II ESI-Q-TOF system set up with Burker Dalotonik Elute UPLC system. Eluted

compounds were ionized in a mass spectrometer, analyzed by mass-to-charge ratio (m/z), and identified based on spectral libraries (20).

2.10. Preparation of C. montanus ~~V. montana~~ Nanoemulsions (NEs)

The ethanol extract was selected for NE formulation to enhance pulmonary delivery based on the high TPC and TFC of this extract as well as its strong antioxidant and antiproliferative activities. This extract was incorporated into an oil-in-water (O/W) NE~~s~~ using the spontaneous emulsification method (21). Briefly, 100 mg of the extract was dissolved in 1 mL of ethanol, and volumes of either 250 µL or 500 µL of this solution were mixed with 2.25 mL or 1.75 mL of ethanol under continuous stirring for 6 minutes at RT. Oleic acid and Tween 20 were then added to the mixture, followed by the dropwise addition of deionized water to facilitate the formation of the NE. The detailed composition of the prepared formulations is provided in Table ~~7~~1. The resulting NEs were sonicated for 30 minutes to ensure transparency and remove any bubbles. The formulations were stored at room temperature (RT) or 4 °C for further stability evaluation. This method allowed for the preparation of stable NEs with varying compositions, which were subsequently characterized for their physicochemical properties and stability. The S4 NE was subjected to thermodynamic stability studies (22).

Table ~~7~~1. The composition of *C. montanus ~~V. montana~~* NEs prepared using the spontaneous emulsification method.

Formulations	Ethanol Extract (µg)	Ethanol (mL)	Tween20 (mL)	Oleic acid (mL)	Deionized water (mL)	Storage condition
**S1**	500	1.75	1.25	0.5	1.00	RT
**S2**	250	2.25	1.50	0.5	0.75	4°C
**S3**	250	2.25	1.25	0.5	1.00	4°C
**S4**	250	2.25	1.25	0.25	1.25	RT
**S5**	250	2.25	1.25	0.5	1.00	RT
**S6**	250	2.25	1.25	0.25	1.25	4°C

2.11. Characterization of C. montanus ~~V. montana~~ NEs

2.11.1. Thermodynamic Stability

Thermodynamic stability was assessed using centrifugation (3500 rpm, 15 min) and heating-cooling cycles (six cycles at 45 °C and 4 °C over 48 hours). Stability was further evaluated with freeze-thaw cycles (-21 °C to 25 °C) over 48 hours.

2.11.2. Physical Stability

Physical stability of NEs was monitored for 60 days at RT and at 4 °C.

2.11.3. pH- and Viscosity Measurements

pH was measured using a Jenway Benchtop pH Meter (Cole-Parmer Ltd, Model 3510, UK). Viscosity was determined with a rotational rheometer (QC Rheolab, Anton Paar, Austria) at 25 °C, with shear rates ranging from 10 to 1000 s^{-1}.

2.11.4. Size Distribution and Zeta Potential

Mean droplet size (MDS), polydispersity index (PDI), and zeta potential (ZP) were measured using dynamic light scattering (DLS) (Nicomp Nano Z3000, Entegris, USA) at 25 °C. Measurements were conducted in triplicate weekly for two months.

2.11.5. Transmission Electron Microscopy (TEM) Analysis

NE morphology was analyzed using a transmission electron microscope (TEM, FEI Morgani 268, Holland). Samples were placed on carbon-coated copper grids, dried for 30 min, and imaged at an accelerating voltage of 120 kV.

3. Results

3.1. Gas Chromatography-Mass Spectrometry (GC-MS) Analysis of C. montanus~~V. montana~~

The hydro-distillation of *C. montanus* ~~*V. montana*~~ yielded a bright yellow essential oil with an intensive characteristic odor. The yield was 0.094% (v/w). GC-MS analysis revealed 31 constituents, with monoterpenes as the dominant chemical class (74.6% of the total composition). Monoterpenes were further categorized into hydrocarbon monoterpenes (37.7%) and oxygenated monoterpenes (36.9%) as shown in Table ~~1~~2. Additionally, oxygenated sesquiterpenes contributed to 14.9%, while miscellaneous compounds accounted for 7.5% of the composition. The most abundant compounds identified in the HD extract were α-phellandrene (28.60%), 1,8-cineole (23.30%), and α-cadinol (11.90%).

Solid-phase micro-extraction (SPME) analysis revealed a higher proportion of monoterpenes (93.0%), distributed as hydrocarbon monoterpenes (50.4%) and oxygenated monoterpenes (42.6%). Sesquiterpenes were detected in significantly lower quantities (1.4%), with hydrocarbon sesquiterpenes comprising 0.6% and oxygenated sesquiterpenes 0.8%. The predominant volatile constituents of the aroma profile were 1,8-cineole (35.60%), sabinene (12.70%), and α-artemisia triene (11.40%).

Table ~~1~~2. Composition of the volatile oils of the fresh aerial parts of *C. montanus* ~~*V. montana*~~ obtained by HD and SPME

No.	KI Lit.*	KI Exp.	Compound	HD %	SPME %
1	923	909	α-Artemisia triene[a]	-	11.40
2	932	933	α- Pinene[a]	00.10	0.40
3	945	965	α- Fenchen[a]	00.20	0.40
4	964	941	Camphene[a]	-	10.00
5	969	1019	Sabinene[a]	-	12.70
6	974	1005	β- Pinene[a]	00.20	5.20

7	988	1016	Myrcene[a]	-	0.10
8	999	1220	Yomogi alcohol[b]	06.00	0.30
9	1002	1063	α-Phellandrene[a]	28.60	0.10
10	1008	1052	Carene[a]	03.00	0.80
11	1014	1181	α-Terpinene[a]	00.90	1.80
12	1020	1172	β- Cymene[a]	00.50	2.20
13	1024	8617	Limonine[e]	-	3.60
14	1025	1031	β-Phellandrene[a]	-	2.10
15	1026	1198	1.8 Cineole[b]	23.30	35.00
16	1034	1225	Santolina alcohol[b]	00.20	2.60
17	1054	1058	Terpinene <γ[a]	-	3.20
18	1065	1252	Sabinene hydrate[b]	00.80	3.80
19	1086	1204	Terpinolene[a]	03.40	-
20	1095	1301	Linalool[b]	01.10	0.10
21	1135	1602	E-Pinocarveol[b]	00.60	0.60
22	1136	1304	E-*p*-Menthenol[e]	01.70	-
23	1141	999	Camphor[b]	00.90	-
24	1160	1378	Pinocarvone[b]	02.70	0.10
25	1165	1665	Borneol[b]	00.50	-
26	1186	1641	α- Terpineol[b]	01.20	0.10
27	1194	1670	Myrtenol[b]	00.20	-
28	1254	1412	Bornyl acetate[e]	00.20	-
29	1277	1641	Ethyl chrysanthemumate[e]	00.20	-
30	1288	1484	Lavandulyl acetate[e]	01.70	-
31	1298	1698	Carvacrol[b]	00.20	-
32	1311	1536	Pinocarvyl acetate[e]	00.10	-
33	1439	1403	Aromadendrene[c]	-	0.30
34	1522	1043	Cadinene[c]	-	0.30
35	1548	1653	α-Cadinol[d]	11.90	0.40
36	1582	1495	Caryophyllene oxide[d]	01.00	0.40
37	1644	1687	α-Muurolol[d]	00.80	-
38	1665	1705	Intermedeol[d]	01.20	-
39	1783	1677	Eudesmol acetate[e]	03.60	-
	**Monoterpenes**			74.6%	93.0%
	Oxygenated monoterpenes[a]			37.7 %	42.6%
	Monoterpene hydrocarbons[b]			36.9%	50.4%

**Sesquiterpenes**	14.9%	1.4%
Oxygenated sesquiterpenes[c]	14.9%	0.8%
Sesquiterpene hydrocarbons[d]	0%	0.6%
[e]Miscellaneous	07.5%	3.6%
Total identified	97.0%	98.0%

*Lit KI: Reported Kovats Index, [a]Oxygenated monoterpenes, [b]Monoterpene hydrocarbons, [c]Oxygenated sesquiterpenes, and [d]Sesquiterpene hydrocarbons.

3.2. Total Phenol (TPC) and Total Flavonoid Contents (TFC) Determinations

The quantitative analysis of TPC and TFC in different solvent extracts of *C. montanus* ~~*V. montana*~~ is summarized in Table ~~2~~3. The ethyl acetate fraction exhibited the highest TPC (47.94 ± 0.32 mg GAE/g), followed by ethanol extract and methanol fraction~~1~~. Similarly, TFC levels were highest in the ethyl acetate fraction (20.34 ± 1.48 mg rutin/g), followed by ethanol extract and chloroform fraction. The aqueous extract displayed the lowest TPC and TFC.

Table ~~2~~3. Total phenol content and total flavonoid content of *C. montanus* ~~*V. montana*~~ extracts

Extract	TPC (GAE mg of gallic acid/g)	TFC (mg of rutin /g)
Ethanol*	20.54 ± 0.41	10.27 ± 0.74
Water*	1.03 ± 0.56	0.12 ± 0.06
Petroleum ether**	1.47 ± 0.34	1.63 ± 0.71
Chloroform**	6.55 ± 0.50	9.71 ± 2.03
Ethyl acetate**	47.94 ± 0.32	20.34 ± 1.48
Methanol**	19.19 ± 0.27	5.28 ± 0.34

Data are shown as the average of triplicates ± SD, n = 3; *Extract; **Fraction.

3.3. Antioxidant Activity

The results indicated that the ethanol extract exhibited the lowest IC_{50} (322.1 ± 39.9 µg/mL), followed by the chloroform - (350.3 ± 68.1 µg/mL) and the petroleum ether- fractions (488.4 ± 109.9 µg/mL). The ethyl acetate fraction showed an IC_{50} of 843.3 ± 171.7 µg/mL, while the methanol fraction had an IC_{50} of 889.9 ± 45.3 µg/mL. The aqueous extract demonstrated the lowest antioxidant activity, with an IC_{50} of 1077.7 ± 60.9 µg/mL.

3.4. Antiproliferative Activity

The cytotoxic effects of the extracts and fractions were evaluated against A549 and H1299 cancer cell lines, as well as against gingival fibroblasts. The chloroform and petroleum ether fractions were excluded from further studies due to their toxicity to normal fibroblast cell lines. The aqueous extract exhibited weak cytotoxicity against the H1299 cell line, with an IC_{50} of 984.4 µg/mL, but showed no activity against the A549 cell line (Table ~~3~~ 4 and Figure 1). In contrast, methanol fraction displayed no cytotoxicity against the A549 or H1299 cancer cell. Ethanol extract and ethyl acetate fraction exhibited strong cytotoxic activity against the A549 and H1299 cell lines without affecting gingival fibroblasts. The IC_{50} for the ethanol extract was 476.5 and 641.2 µg/mL against the A549 and H1299 cell lines, respectively. Similarly, the IC_{50} for the ethyl acetate fraction was 468.0 and 877.6 µg/mL against the A549 and H1299 cell lines, respectively.

Table ~~3~~4. The IC_{50} (µg/mL) for different extracts of *C. montanus* ~~*V. montana*~~ tested on three cell lines (A549, H1299, and Fibroblasts).

C. montanus ~~*V. montana*~~ extracts	A549 cancer cell (µg/mL)	H1299 cancer cell (µg/mL)	Fibroblast (µg/mL)
Ethyl acetate	468.035	877.55	Nontoxic
Ethanol	476.545	641.175	Nontoxic
Methanol	Nontoxic	Nontoxic	Nontoxic
Aqueous extract	Nontoxic	984.4	Nontoxic

Data are represented as mean ± SD, n=3.

Figure 1. Antiproliferative effect of *C. montanus* ~~*V. montana*~~ extracts on A) A549; B) H1299; and C) fibroblasts. Data are represented as mean ± SEM, n=3.

3.5. Liquid Chromatography-Mass Spectrometry (LC-MS)

The active ethanol extract and ethyl acetate fraction, were subjected to LC-MS analysis to identify the phytochemical composition to link the identified compounds with their cytotoxic activities. The LC-MS analysis of the ethanol extract revealed the identification of 14 compounds (Table ~~4~~5).

Table ~~4~~5. Identified phytochemicals in the ethanol and ethyl acetate extracts of *C. montanus* ~~*V. montana*~~ by LC-MS, expressed as % within identified phytochemicals

Rt	Phytochemicals	Molecular Formula	Ethanol extract	Ethyl-acetate extract
1.9	2,5-dihydroxybenzoic acid	$C_7H_6O_4$	-	10.6
3.1	Chlorogenic acid	$C_{16}H_{18}O_9$	22.5	16.7
3.5	Caffeic acid	$C_9H_8O_4$	3.5	20.6
5.7	Scopoletin	$C_{10}H_8O_4$	1.1	0.1

5.8	Taxifolin	$C_{15}H_{12}O_7$	-	3.9
6.0	Spiraeoside	$C_{21}H_{20}O_{12}$	8.9	8.9
6.4	Aesculetin	$C_9H_7O_4$	1.3	-
6.2	Rosmarinic acid	$C_{18}H_{16}O_8$	1.0	0.1
6.3	5-Glc tricin	$C_{23}H_{24}O_{12}$	-	3.2
6.7	Quercetin	$C_{21}H_{20}O_{11}$	-	7.5
7.0	Myricetin	$C_{15}H_{10}O_8$	0.9	0.1
7.3	Aesculetin	$C_9H_6O_4$	-	0.1
8.9	3,6,2,3-tetrahydroxyflavone	$C_{15}H_{10}O_6$	-	2.4
9.3	Quercetin-4′-O-malonylglucoside	$C_{24}H_{22}O_{15}$	1.5	0.3
9.5	6-methoxyluteolin	$C_{16}H_{12}O_7$	0.2	2.2
10.4	Kaempferol	$C_{15}H_{10}O_6$	1.7	-
10.8	Silymarin	$C_{25}H_{22}O_{10}$	0.5	0.1
11.0	5,6,4-trihydroxy-7.3-dimethoxy flavone	$C_{17}H_{14}O_7$	0.9	0.1
11.1	Sorbifolin	$C_{16}H_{12}O_6$	14.4	-
13.3	Nevadensin	$C_{18}H_{16}O_7$	41.6	23.1

Seventeen compounds were identified in the ethyl acetate fraction using LC-MS. Similar to the ethanol extract, nevadensin and chlorogenic acid were identified as the major compounds in this fraction, along with caffeic acid. The detailed composition is summarized in Table 45.

3.6. Characterization of Ethanol Extract-Loaded Nanoemulsion (NE)

3.6.1. Centrifugation

The stability of the S4 NE was evaluated through centrifugation at 3500 rpm for 15 min. post-centrifugation, all formulations remained homogeneous and transparent, confirming their stability.

3.6.2. Heating/Cooling Cycles

To further assess the stability, S4 NE was subjected to six heating and cooling cycles and three freezing and thawing cycles. No signs of instability, such as cracking, creaming, or separation, were observed throughout these cycles, demonstrating a stable NE.

3.6.3. Morphology and Size

The TEM images of S4 NE revealed that the droplets were uniformly distributed, distinct, and spherical in shape, with no aggregation. The droplet sizes measured by TEM were 12.49 nm and 20.14 nm as shown in Figure 2, which aligned closely with the results obtained using a nanosizer of 13.6 ± 0.5 nm.

Figure 2. TEM image of S4 NE of spherical-shaped droplets.

3.6.4. pH Measurement

The initial pH measurement of S4 NE was 5.39 ± 0.06. After 1 and 2 months of storage at RT (25 ± 2 °C), the pH was 5.36 ± 0.04 and 5.02 ± 0.15. It has been shown that the pH of liquid preparations intended for pulmonary delivery should ideally fall between 3.0 and 8.5, corresponding to the pH of lung fluid (23). Hence, the pH values were within the acceptable range specified by the European Pharmacopeia for pulmonary medicines, making these formulations suitable for lung delivery(23).

3.6.5. Viscosity Measurement

The viscosity curve of S4 NE demonstrated a shear-thinning behavior, as illustrated in Figure 3. This behavior indicates that the material becomes less viscous as the shear rate increases.

Figure 3. Viscosity curve of S4 NE. Data is presented as mean ± SD (n=3).

3.6.6. Physical Stability

The physical evaluation of S4 NE after two months of storage at RT and 4 °C revealed that the formulation remained transparent and uniform with no color change.

3.6.7. Stability of Droplet Size, Polydispersity Index (PDI), and Zeta Potential (ZP) Measurements

The stability of S4 NE was evaluated by monitoring changes in mean droplet size (MDS) and PDI over a two-month period under two storage conditions: RT and 4 °C. Initial measurements showed that the droplet size and PDI were within the acceptable range for nanoemulsions <100 nm(21).

Over time, both parameters exhibited a gradual increase, but the droplet size remained below 100 nm, indicating good stability. Detailed numerical results for MDS and PDI at each time point are provided in Table 56.

The stability studies confirmed that S4 NE maintained its physicochemical properties, including droplet size, PDI, and zeta potential, within acceptable ranges over two months of storage at both, RT and 4 °C. The gradual increase in droplet size and PDI did not compromise the nanosized nature of the formulation, while the zeta potential measurements indicated sufficient stability despite being below the conventional threshold. These findings highlight the robustness of S4 NE for potential therapeutic applications (24, 25).

Table 56. The stability measurements of MDS, PDI, and ZP of S4 NEs at RT and 4 ºC.

Time	Stored at RT	Stored at 4 ºC

	MDS	PDI	ZP	MDS	PDI	ZP
**Initial**	13.6±0.5	0.1±0.05	-1.7±0.6	13.6±0.5	0.1±0.05	-1.7±0.6
**1 week**	13.9±0.06	0.2±0.02	-6.7±1.7	13.4±0.2	0.2±0.01	-3.5±1.7
**2 weeks**	16.3±0.1	0.3±0.01	-6.1±0.4	12.6±0.6	0.1±0.01	-6.0±0.4
**3 weeks**	20.5±0.33	0.3±0.005	-6.8±0.4	23.9±0.5	0.3±0.01	-10.2±1.6
**1 month**	25.7±0.73	0.3±0.03	-6.6±3.0	17.8±0.2	0.2±0.04	-12.2±0.04

Data are presented as mean ± SD (n=3).

3.7. Cytotoxicity Evaluation of C. montanus ~~V. montana~~ NE

The cytotoxic effects of *C. montanus* ~~*V. montana*~~ NE were evaluated against A549 and H1299 cancer cell lines and normal fibroblasts using the MTT assay (Table ~~6~~ 7 and Figure 4). Experiments were performed in triplicate, and results are expressed as mean ± SD. The IC_{50} values for *C. montanus* ~~*V. montana*~~ NE are given in Table ~~6~~7. A549 and H1299 cells exhibited similar sensitivity to the NE, while no cytotoxicity was observed against fibroblasts, indicating its selectivity for cancer cells and safety for normal cells.

Table ~~6~~7. The IC_{50} (µg/mL) for *C. montanus* ~~*V. montana*~~ NE tested on A549, H1299, and fibroblasts cells.

	A549 (µg/mL)	H1299 (µg/mL)	Fibroblast (µg/mL)
C. montanus ~~***V. montana***~~ **extract**	383.5 ± 7.061	343 ± 6.24	1038.3 ± 62.11

Data are represented as mean ± SD, n=3.

Figure 4. Cytotoxicity profile and IC_{50} of *C. montanus* ~~*V. montana*~~ NE when used to treat **A)** A549; **B)** H1299; and **C)** Fibroblasts cell lines. The X-axis shows the log concentration in µM/mL, and the Y-axis shows the percentage of inhibited cells.

4. Discussion

The phytochemical analysis of *C. montanus* ~~*V. montana*~~ revealed a diverse composition of volatile and non-volatile bioactive compounds. A recent study by Abu-Darwish et al. (2022) identified sesquiterpene hydrocarbons (47.97%) and oxygenated sesquiterpenes (35.32%) as the major volatile compounds in the aroma profile of wild-growing *C. montanus* ~~V. montana~~ collected from Jordan (13). Their analysis utilized dried plant material, whereas the present study employed fresh plant material for SPME analysis. These differences may be attributed to environmental factors, such as soil characteristics, temperature variations, and other climatic conditions, in addition to laboratory methodologies. Notably, Abu-Darwish collected plant material from the Mediterranean biogeographic zone of Jordan, while in this study, mature plants were sourced from the Irano-Turanian biogeographic zone (13, 26).

The findings highlight the impact of extraction techniques on the volatile profile of *C. montanus*~~V. montana~~. Hydro-distillation yielded a broader spectrum of volatile compounds, particularly sesquiterpenes, while

SPME selectively enriched monoterpenes, predominantly oxygenated forms. The significant abundance of α-phellandrene (28.60%) and 1,8-cineole (23.30%) suggests their pharmacological relevance to the ethanol extract, given their solubility properties (27, 28).

The TPC and TFC analyses demonstrated the influence of solvent polarity on the extraction efficiency of bioactive compounds. Ethyl acetate, a semi-polar solvent, yielded the highest TPC (47.94 ± 0.32 mg GAE/g) and TFC (20.34 ± 1.48 mg rutin/g), followed by ethanol, which also exhibited considerable extraction efficiency (TPC: 20.54 ± 0.41 mg GAE/g; TFC: 10.27 ± 0.74 mg rutin/g). In contrast, water and non-polar solvents like petroleum ether showed significantly lower extraction capacities. The strong correlation between TPC and TFC suggests that the polyphenolic fraction of *C. montanus* ~~V. montana~~ is predominantly composed of flavonoids, which are known for their antioxidant and cytotoxic activities (29, 30). These results, in conjunction with antioxidant activity assessments, underscore the phytochemical richness of *C. montanus* ~~V. montana~~ and its potential as a therapeutic botanical resource, particularly when extracted using ethyl acetate and ethanol.

Cytotoxicity evaluations revealed that the ethanol extract and ethyl acetate fraction exhibited promising selective cytotoxic effects on cancer cell lines while sparing normal fibroblasts. Recent studies further support the pharmacological potential of *C. montanus*~~V. montana~~. Elhady et al. reported that *C. montanus* ~~V. montana~~ extract and its isolated flavonoid, jaceidin, exhibited strong cytotoxic activity against HepG2 and MCF-7 cells, with *in vivo* studies demonstrating tumor-suppressing effects via VEGF inhibition and oxidative stress modulation (8). Additionally, Aidy et al. found that the methanol and petroleum ether extracts of *C. montanus*~~V. montana~~ displayed significant cytotoxicity against colorectal cancer cell lines and promoted colorectal cancer stem cell differentiation through modulation of key genes and microRNA expressions (9).

The ethanol extract's major identified compound, nevadensin (5,7-dihydroxy-6,8,4′-trimethoxyflavone), is known for its diverse biological activities, including hypotensive, anti-inflammatory, antimicrobial, and anticancer properties (31). Studies have identified nevadensin as a selective inhibitor of human carboxylesterase 1 and topoisomerases TOPO I and TOPO IIα using in vitro models (32, 33).

Chlorogenic acid, a widely distributed phenolic acid, exhibits multiple biological effects, including hepatoprotective, antimicrobial, immunomodulatory, antioxidant, antidiabetic, and anticancer properties. Studies have demonstrated its role in cancer prevention through mechanisms such as inhibition of cell division, angiogenesis suppression, cell cycle arrest, and apoptosis induction, supported by in vitro, in vivo, and clinical studies (34, 35). Notably, chlorogenic acid has been approved by the China Food and Drug Administration as a natural anticancer drug (34).

Sorbifolin (Scutellarin-7-methylether), another major compound in the ethanol extract, is recognized for its antioxidant, anticancer, antimicrobial, anti-inflammatory, and antidiabetic activities(36). *In silico* studies by Shah and Kin concluded that sorbifolin is a potent candidate for Ewing sarcoma treatment (37).

These findings suggest that the identified phytochemicals, particularly flavonoids, play a significant role in the cytotoxic activity observed in the ethanol extract. Given the well-established antioxidant properties of phenolics and flavonoids, their ability to neutralize oxygen radicals *via* redox mechanisms may contribute to their antiproliferative effects (38). Further research is warranted to elucidate their specific mechanisms of action.

To enhance pulmonary delivery for lung carcinoma treatment, nanoemulsions (NEs) were developed. The optimized S4 NE formulation demonstrated excellent stability and an appropriate droplet size for pulmonary administration. Cytotoxicity evaluations revealed that *C. montanus* ~~V. montana~~ NE selectively targeted cancer cells (A549 and H1299) while sparing normal fibroblasts, supporting its potential as a safe and effective anticancer formulation. These findings emphasize the therapeutic promise of *C. montanus* ~~V. montana~~ NE for targeted cancer therapy, warranting further investigation into its mechanisms of action and *in vivo* efficacy.

5. Conclusions

This comprehensive study on *C. montanus* ~~*V. montana*~~ highlights its significant phytochemical diversity and pharmacological potential. The GC-MS analysis revealed that HD hydro-distillation, produces a more diverse composition rich in sesquiterpenes, while SPME was selectively enriched with monoterpenes. The quantitative analysis of TPC and TFC demonstrated that ethyl acetate fraction and ethanol extract contain large amounts of these bioactive compounds, aligning with their strong antioxidant and cytotoxic activities. Ethanol extract and ethyl acetate fraction showed promising selective cytotoxicity against A549 and H1299 cancer cell lines, without affecting normal fibroblasts, underlining their potential for therapeutic applications.

LC-MS analysis identified key phytochemicals, including nevadensin and chlorogenic acid, which likely contribute to the observed bioactivities. The formulation of an ethanol extract-loaded NEs further enhanced the delivery potential of *C. montanus* ~~*V. montana*~~ extracts, with S4 NE demonstrating excellent stability, suitable droplet size, and pH for pulmonary delivery. The cytotoxicity evaluation of the NE confirmed its selective anticancer activity, with no adverse effects on normal cells.

Overall, this study underscores the therapeutic potential of *C. montanus* ~~*V. montana*~~ as a rich source of bioactive compounds with antioxidant, cytotoxic, and anticancer properties. The findings suggest that *C. montanus* ~~*V. montana*~~ extracts, particularly when formulated as NEs, are promising natural therapeutic agents, especially in cancer treatment. Future studies should focus on elucidating the mechanisms of action of the identified compounds and exploring their *in vivo* efficacy and safety profiles.

Author Contributions: Conceptualization, N.H. and F.A.; methodology, N.H., H.A, H.S. and I.A; software, N.H., M.W. and E.A.; validation, N.H., R.H. and M.W.; formal analysis, N.H.; investigation, N.H. and F.A.; resources, N.H.; data curation, N.H.; writing—original draft preparation, H.A. and H.S.; writing—review and editing, N.H., R.H. M.W. and F.A.; visualization, N.H.; supervision, N.H. and F.A.; project administration, N.H.; funding acquisition, N.H.; Plant material collection and identification, F.A and I.A. All authors have read and agreed to the published version of the manuscript.

Funding: This research was funded by Deanship of Scientific Research and Innovation at Al-Zaytoonah University of Jordan (2022-2023/07/23).

Data Availability Statement: All data generated or analyzed during this study are included in this published article.

Conflicts of Interest: The authors declare no conflicts of interest. The funders had no role in the design of the study; in the collection, analyses, or interpretation of data; in the writing of the manuscript; or in the decision to publish the results.

Abbreviations

The following abbreviations are used in this manuscript:

DLS	Dynamic Light Scattering
GC-MS	Gas Chromatography-Mass Spectrometry
HD	HydroDistillation
LC-MS	Liquid Chromatography-Mass Spectrometry
MDS	Mean Droplet Size
NEs	Nanoemulsions
PDI	PolyDispersity Index
SPME	Solid-Phase Micro-Extraction
TEM	Transmission Electron Microscopy
TFC	Total Flavonoid Content
TPC	Total Phenol Content
ZP	Zeta Potential

References

1. Hegazy MF, Matsuda H, Nakamura S, Hussein TA, Yoshikawa M, Paré PW. Chemical constituents and their antibacterial and antifungal activity from the Egyptian herbal medicine Chiliadenus montanus. Phytochemistry. 2014;103:154-61.
2. Castillo LV, García FZ, Arnáez CZ. The Genus Chiliadenus: A Comprehensive Review of Taxonomic Aspects, Traditional Uses, Phytochemistry and Pharmacological Activities. Plants. 2025;14(2):205.
3. Afifi FÜ, Al-Khalil S, Abdul-Haq BK, Mahasneh A, Al-Eisawi DM, Sharaf M, et al. Antifungal flavonoids from Varthemia iphionoides. Phytotherapy Research. 1991;5(4):173-5.
4. Afifi F, Aburjai T. Antiplatelet activity of Varthemia iphionoides. Fitoterapia. 2005;75:629-33.
5. Al-Dabbas M, Fumio H, Abdelgaleil S, Suganuma T, Akiyama K, Hayashi H. Antibacterial activity of an eudesmane sesquiterpene isolated from common Varthemia, Varthemia iphionoides. Journal of Ethnopharmacology. 2005;97:237-40.
6. Soliman FM, Moussa MY, Abdallah H, Othman SM. Cytotoxic activity of flavonoids of Jasonia Montana Vahl. (Botsch). (Astraceae) growing in Egypt. Australian Journal of Basic and Applied Sciences, 2009;3(1):148-52.
7. Hussein MA, Abdel-Gawad SM. Protective effect of Jasonia montana against ethinylestradiol-induced cholestasis in rats. Saudi pharmaceutical journal : SPJ : the official publication of the Saudi Pharmaceutical Society. 2010;18(1):27-33.
8. Elhady SS, Eltamany EE, Shaaban AE, Bagalagel AA, Muhammad YA, El-Sayed NM, et al. Jaceidin Flavonoid Isolated from Chiliadenus montanus Attenuates Tumor Progression in Mice *via* VEGF Inhibition: In Vivo and In Silico Studies. Plants. 2020;9(8).
9. Aidy EA, Fathy Z, Ahmed WA, El-Gendy S, Abou-Bakr A, Omran M, et al. Jasonia montana Extracts Stimulate Cell Differentiation in HCT-116 and Caco-2 Cell Lines %J Egyptian Journal of Chemistry. 2022;65(12):165-74.
10. El-Bsoumy E, Habib E, Ibrahim A, ahmed s. Phytochemical constituents and biological activities of Jasonia montana (Asteraceae). Records of Pharmaceutical and Biomedical Sciences. 2021;5:1-11.

11. Abu-Darwish D, Shibli R, Al-Abdallat AM. Phenolic Compounds and Antioxidant Activity of Chiliadenus montanus (Vhal.) Brullo. grown in vitro. Jordan Journal of Pharmaceutical Sciences. 2024;17(3):611-28.
12. shaaban a, El Tamany E, Ayyad S, ahmed s. Genus chiliadenus is a unique supplier of several chemical flocks: A review. Records of Pharmaceutical and Biomedical Sciences. 2021;5:20-40.
13. Abu-Darwish D, Shibli R, Al-Abdallat AM. In Vitro Cultures and Volatile Organic Compound Production in Chiliadenus montanus (Vhal.) Brullo. Plants. 2022;11(10).
14. Al-Eisawi. Flora of Jordan Checklist Revised Edition 1. University of Jordan: The University of Jordan Press. Amman, Jordan; 2013.
15. Ferhat MA, Meklati BY, Smadja J, Chemat F. An improved microwave Clevenger apparatus for distillation of essential oils from orange peel. Journal of Chromatography A. 2006;1112(1-2):121-6.
16. Afifi FU, Kasabri, V., & Abaza, I. M. <GC-MS composition and antiproliferative activity of Inula graveolens (L.).pdf>. Arabian Journal of Medicinal and Aromatic Plants. 2015;1:57-66.
17. Adams R. Identification of Essential Oil Components by Gas Chromatography/Quadrupole Mass Spectroscopy. Carol Stream. 2005;16:65-120.
18. Aboalhaija NH, Syaj H, Afifi F, Sunoqrot S, Al-Shalabi E, Talib W. Chemical Evaluation, *In Vitro* and *In Vivo* Anticancer Activity of Lavandula angustifolia Grown in Jordan. Molecules 2022;27(18).
19. Aboalhaija A, Amro R, Abaza I, Khalil E, Al-Aboudi A, Abu Zarga M, et al. Schinus molle L. Collected from Jordan and Turkey: Essential Oil Composition and Anticholinesterase Activity. Journal of Essential Oil Bearing Plants. 2019;22:704-16.
20. Mohammad AA-M, Nour HA, Ismail FA, Mohammad HK, Malek AZ, Fatma UA. Chromatographic Analysis (LC-MS and GC-MS), Antioxidant Activity, Total Phenol and Total Flavonoid Determination of Ononis natrix L. Grown in Jordan. Jordan Journal of Chemistry (JJC). 2021;16(1):31-9.
21. Hamed R, Basil M, AlBaraghthi T, Sunoqrot S, Tarawneh O. Nanoemulsion-based gel formulation of diclofenac diethylamine: design, optimization, rheological behavior and in vitro diffusion studies. Pharmaceutical Development and Technology. 2016;21(8):980-9.
22. Ahmadi O, Jafarizadeh-Malmiri H. Intensification process in thyme essential oil nanoemulsion preparation based on subcritical water as green solvent and six different emulsifiers. Green Processing and Synthesis 2021; 10(1):430-9.
23. Weber S, Zimmer A, Pardeike J. Solid Lipid Nanoparticles (SLN) and Nanostructured Lipid Carriers (NLC) for pulmonary application: a review of the state of the art. European Journal of Pharmaceutics and Biopharmaceutics 2014;86(1):7-22.
24. Hamed R, Abu Alata Wa, Abu-Sini M, Abulebdah DH, Hammad AM, Aburayya R. Development and Comparative Evaluation of Ciprofloxacin Nanoemulsion-Loaded Bigels Prepared Using Different Ratios of Oleogel to Hydrogels. Gels, 2023;9(7):592.
25. Xu XY, Yu Z, Zhu Y, Wang B. Effect of sodium oleate adsorption on the colloidal stability and zeta potential of detonation synthesized diamond particles in aqueous solutions. Diamond and Related Materials - DIAM RELAT MATER. 2005;14.
26. C. P. Biogeography. In M. Ababsa (Ed.), Atlas of Jordan. In: l'Ifpo Pd, editor.
Atlas of Jordan. Beyrout, Lebanon Publi-cation sur OpenEdition Books; 2013. p. 77-87.
27. Shiekh RAE, Atwa AM, Elgindy AM, Mustafa AM, Senna MM, Alkabbani MA, et al. Therapeutic applications of eucalyptus essential oils. Inflammopharmacology. 2025;33(1):163-82.
28. de Cássia da Silveira e Sá R, Andrade LN, de Sousa DP. A review on anti-inflammatory activity of monoterpenes. Molecules. 2013;18(1):1227-54.
29. Ahmed SI, Hayat MQ, Tahir M, Mansoor Q, Ismail M, Keck K, et al. Pharmacologically active flavonoids from the anticancer, antioxidant and antimicrobial extracts of Cassia angustifolia Vahl. BMC Complementary and Alternative Medicine. 2016;16(1):460.

30. Kumar S, Pandey AK. Chemistry and biological activities of flavonoids: an overview. The Scientific World Journal. 2013;2013:162750.
31. Brahmachari G. Nevadensin: Isolation, chemistry and bioactivity. International Journal of Green Pharmacy. 2010;4(4).
32. Wang YQ, Weng ZM, Dou TY, Hou J, Wang DD, Ding LL, et al. Nevadensin is a naturally occurring selective inhibitor of human carboxylesterase 1. International Journal of Biological Macromolecules. 2018;120(Pt B):1944-54.
33. Müller L, Schütte LRF, Bücksteeg D, Alfke J, Uebel T, Esselen M. Topoisomerase poisoning by the flavonoid nevadensin triggers DNA damage and apoptosis in human colon carcinoma HT29 cells. Archives of Toxicology. 2021;95(12):3787-802.
34. Gupta A, Atanasov AG, Li Y, Kumar N, Bishayee A. Chlorogenic acid for cancer prevention and therapy: Current status on efficacy and mechanisms of action. Pharmacological Research. 2022;186:106505.
35. Singh S, Varshney M. Exploring the Pharmacological Potential of Chlorogenic acid as an Anti-Cancer Agent and a Call for Advance Research. Combinatorial Chemistry & High Throughput Screening. 2025; 28(12).
36. Belmehdi O, Mssillou I, Khalid A, Abdalla AN, Almalki M, Alqurashi RS, et al. Unveiling the Biological Activities, Pharmacological Potential, and Health Benefits of Sorbifolin: A Comprehensive Review. Chemistry & Biodiversity 2025;22(2):e202401463.
37. Shah FH, Kim SJ. Inhibitors of EYA3 Protein in Ewing Sarcoma. Asian Pacific Journal of Cancer Prevention : APJCP. 2022;23(5):1539-45.
38. Althaher AR, Shehabi RF, Ameen HH, Awadallah MW, Mastinu A. Calamintha incana Methanolic Extract: Investigation of Phytochemical Composition and Antioxidant and Antibacterial Activities. Journal of Food Biochemistry 2024;2024(1):6634969.

9-May-2025

Re: Manuscript ID: PONE-D-25-17902

Dear Dr. Šiler,

We sincerely thank you and the reviewers for your constructive and insightful comments on our manuscript. We appreciate the opportunity to revise and resubmit our work.

We have carefully considered each comment and revised the manuscript accordingly. In brief:

1. Please update the Latin name of the study species. *Varhemia montana* is actually a synonym of *Chiliadenus montanus* (Vahl) Brullo (e.g., https://powo.science.kew.org/taxon/urn:lsid:ipni.org:names:257204-1).
   The Latin name of the plant species has been updated to *Chiliadenus montanus (Vahl) Brullo*, in accordance with current taxonomic classifications.

2. The whole first paragraph of the introductory section needs thorough revision, as the taxonomy of the former genus *Varhemia* has been changed since 2018.

   The first paragraph of the introduction has been thoroughly revised to reflect this change.

3. Please add line and page numbers to facilitate the future review process.
   Line and page numbers have been added throughout the manuscript to aid future review.
4. Please explain letters in superscripts in Table 2.

   The superscript letters in Table 2 have been clearly explained in a new footnote to enhance clarity.

5. Figure Reorganization: As per PLOS ONE's figure formatting guidelines.

   We have uploaded updated versions of all figures. Figure 1a–c has been split into three separate figures to meet the requirements. We have also split Figure 4 as necessary and uploaded the revised versions accordingly.

We hope the revised version meets your expectations and the requirements of PLOS ONE. Thank you again for your time and consideration.

Nour Aboalhaija, PhD
Assistant Professor of Phytochemistry
Department of Pharmacy
Faculty of Pharmacy
Al-Zaytoonah University of Jordan
Mobile: +962-79-6304081
E-mail: n.aboalhaija@zuj.edu.jo

Google Scholar: https://scholar.google.com/citations?user=CBVZdtcAAAAJ&hl=en
ResearchGate: https://www.researchgate.net/profile/Nour-Aboalhaija
