# Supplementary material for: Phytochemical characterization, anticancer potential, and nanoemulsion-based delivery of Chiliadenus montanus
Source: PLoS One. 2025 Sep 26;20(9):e0333482. doi: 10.1371/journal.pone.0333482 (PMC12469210; doi:10.1371/journal.pone.0333482)
Supplement: Attachment — Submitted filename: Response to Reviewers.docx [file pone.0333482.s004.docx]

9-May-2025

Re: Manuscript ID: PONE-D-25-17902

Dear Dr. Šiler,

We sincerely thank you and the reviewers for your constructive and insightful comments on our manuscript. We appreciate the opportunity to revise and resubmit our work.

We have carefully considered each comment and revised the manuscript accordingly. In brief:

1. Please update the Latin name of the study species. *Varhemia montana* is actually a synonym of *Chiliadenus montanus* (Vahl) Brullo (e.g., <https://powo.science.kew.org/taxon/urn:lsid:ipni.org:names:257204-1>).

The Latin name of the plant species has been updated to *Chiliadenus montanus (Vahl) Brullo*, in accordance with current taxonomic classifications.

2. The whole first paragraph of the introductory section needs thorough revision, as the taxonomy of the former genus *Varhemia* has been changed since 2018.

The first paragraph of the introduction has been thoroughly revised to reflect this change.

3. Please add line and page numbers to facilitate the future review process.

Line and page numbers have been added throughout the manuscript to aid future review.

4. Please explain letters in superscripts in Table 2.

The superscript letters in Table 2 have been clearly explained in a new footnote to enhance clarity.

5. Figure Reorganization: As per PLOS ONE’s figure formatting guidelines.

We have uploaded updated versions of all figures. Figure 1a–c has been split into three separate figures to meet the requirements. We have also split Figure 4 as necessary and uploaded the revised versions accordingly.

We hope the revised version meets your expectations and the requirements of PLOS ONE. Thank you again for your time and consideration.

Nour Aboalhaija, PhD

Assistant Professor of Phytochemistry

Department of Pharmacy

Faculty of Pharmacy

Al-Zaytoonah University of Jordan

Mobile: +962-79-6304081

E-mail: [n.aboalhaija@zuj.edu.jo](mailto:suhair.sunoqrot@zuj.edu.jo)

Google Scholar: <https://scholar.google.com/citations?user=CBVZdtcAAAAJ&hl=en>

ResearchGate: <https://www.researchgate.net/profile/Nour-Aboalhaija>
