# Supplementary material for: Phytochemical characterization, anticancer potential, and nanoemulsion-based delivery of Chiliadenus montanus
Source: PLoS One. 2025 Sep 26;20(9):e0333482. doi: 10.1371/journal.pone.0333482 (PMC12469210; doi:10.1371/journal.pone.0333482)
Supplement: Attachment — Submitted filename: Reviewers comments.docx [file pone.0333482.s003.docx]

This study addresses an interesting and timely topic. The obtained results are significant and may benefit future research. However, before publishing this work, it is necessary to make certain corrections and clarify some doubts. I hope that the following comments will contribute to improving the quality of the manuscript:

In the Abstract, you say: „Spontaneous emulsification was used to formulate ethanolic NEs, with S3, S4, S5, and S6 demonstrating thermodynamic stability, suitable pulmonary pH, and droplet sizes below 200 nm.”… but in the Material and Method section, you indicate that further analyses of thermodynamic stability were done on S4. Please additionally explain what was done and for which NE.

Page 12, line 127: Indicate which cancer cell lines (lung cancer) were used in the study.

Page 12, line 136: Add the used concentration of MT reagent.

Page 13, line 127: Add an explanation for S4 selection for thermodynamic studies.

Page 13, line 165: Specify which physical characteristics are monitored.

In the Materials and Methods, rotate the subheadings 2.11.2 and 2.11.3 so that it is displayed the same as in the Results (or do the rotation in the Results).

Page 14, line 185: „hydrocarbon monoterpenes (37.7%) and oxygenated monoterpenes (36.9%) as shown in Table 2.”… There is a mistake here or in the table regarding the values.

Page 14, line 193: “1,8-cineole (35.60%), sabinene (12.70%), and α-artemisia triene (11.40%). “…Is it necessary to capitalise compound names as written in Table 2? If so, implement the changes throughout the entire manuscript.

Same line: “1,8-cineole (35.60%)” …value does not correspond to value from Table 2.

In the text of the manuscript, you use the term aqueous extract (Page 16, line 202), but in the tables and figures, you use the term water extract. Use one term and make the appropriate changes. Also, the order in which extracts are displayed is not uniform in tables and graphs. Please correct it. Apply the selected term and order of extracts to the figures, also.

Results for Antioxidant activity are not displayed. You could insert them into Table 3.

The plant name remained unchanged on the figures.

The Discussion section should be expanded by comparing the obtained results with the literature data, as well as emphasising the significance of the results of this work. For example, the results of NE cytotoxicity indicate a significantly reduced IC_50_ value of the used NE compared to the corresponding extract, but this is not emphasised or interpreted anywhere in the work.

Recent investigations are focused on *In vitro* callus induction, which generally allows for enhanced production of certain plant extract components, thereby contributing to their enhanced efficacy. It would be useful to mention this in combination with the spontaneous emulsification of the extract as a suggestion for future research. Here are some proposed references:

Abu-Darwish, D., Shibli, R., & Al-Abdallat, A. M. (2022). In vitro cultures and volatile organic compound production in chiliadenus montanus (vhal.) brullo. *Plants*, *11*(10), 1326.

Abu-Darwish, D., Shibli, R., & Al-Abdallat, A. M. (2024). Phenolic Compounds and Antioxidant Activity of Chiliadenus montanus (Vhal.) Brullo. grown in vitro. *Jordan Journal of Pharmaceutical Sciences*, *17*(3), 611-628.
