# Supplementary material for: Phytochemical characterization, anticancer potential, and nanoemulsion-based delivery of Chiliadenus montanus
Source: PLoS One. 2025 Sep 26;20(9):e0333482. doi: 10.1371/journal.pone.0333482 (PMC12469210; doi:10.1371/journal.pone.0333482)
Supplement: Attachment — Submitted filename: Response_to_Reviewers_auresp_2.docx [file pone.0333482.s005.docx]

September 12, 2025

**Manuscript ID:** PONE-D-25-17902R1
**Title:** *Phytochemical Characterization, Anticancer Potential, and Nanoemulsion-Based Delivery of Chiliadenus montanus*

Dear Dr. Šiler,
We sincerely thank you and the reviewers for the constructive comments and valuable feedback that have helped us improve the quality of our manuscript. We have revised the manuscript accordingly, and all modifications are highlighted in the marked-up version (Revised Manuscript with Track Changes). Below, we provide a detailed, point-by-point response to each comment.

**Editor’s Comments**

**==============================
The authors need to provide additional clarification of the methodology used and to add the missing results on antioxidant activity. The Discussion section should be thoroughly revised by comparing the obtained results with those published earlier, as suggested by Reviewer #2. Regarding the Reviewer #2's comment on the capitalization of compound names, please keep them non-capitalized in the text and capitalized in Table 2. Please provide thorough feedback with point-by-point response to reviewers' comments. Note that PLOS ONE do not copyedit the text before publishing. Therefore, I suggest the authors having it thoroughly checked by a native English speaker or a professional editing agency to polish the language. Please be aware the additional reviewers' comments provided in the attached files. 
==============================**

**Please submit your revised manuscript by Oct 18 2025 11:59PM. If you will need more time than this to complete your revisions, please reply to this message or contact the journal office at plosone@plos.org. When you're ready to submit your revision, log on to https://www.editorialmanager.com/pone/ and select the 'Submissions Needing Revision' folder to locate your manuscript file.

Please include the following items when submitting your revised manuscript:**

- **A rebuttal letter that responds to each point raised by the academic editor and reviewer(s). You should upload this letter as a separate file labeled 'Response to Reviewers'.**
- **A marked-up copy of your manuscript that highlights changes made to the original version. You should upload this as a separate file labeled 'Revised Manuscript with Track Changes'.**
- **An unmarked version of your revised paper without tracked changes. You should upload this as a separate file labeled 'Manuscript'.**

**If you would like to make changes to your financial disclosure, please include your updated statement in your cover letter. Guidelines for resubmitting your figure files are available below the reviewer comments at the end of this letter.

If applicable, we recommend that you deposit your laboratory protocols in protocols.io to enhance the reproducibility of your results. Protocols.io assigns your protocol its own identifier (DOI) so that it can be cited independently in the future. For instructions see: https://journals.plos.org/plosone/s/submission-guidelines#loc-laboratory-protocols. Additionally, PLOS ONE offers an option for publishing peer-reviewed Lab Protocol articles, which describe protocols hosted on protocols.io. Read more information on sharing protocols at https://plos.org/protocols?utm_medium=editorial-email&utm_source=authorletters&utm_campaign=protocols.

We look forward to receiving your revised manuscript.**

**Kind regards,**

**Branislav T. Šiler, Ph.D.
Academic Editor
PLOS ONE**

**Comment 1:** **Additional clarification of the methodology used.**
Response: We have expanded the Materials and Methods section to provide full details of the GC-MS, LC-MS, and stability monitoring protocols, including apparatus specifications, operating parameters, and equations used for calculations.

**Comment 2:** **Add the missing results on antioxidant activity.**
Response: We respectfully note that the results on antioxidant activity were originally presented in Section 3.3 (Antioxidant Activity) in textual form. In the revised version, these results have been consolidated into Table 4 and are further elaborated in both the Results and Discussion sections.

**Comment 3:** **The Discussion section should be thoroughly revised by comparing the obtained results with those published earlier.**

Response: We have revised the Discussion section to compare our findings with published studies, highlighting the novelty and significance of our results.

**Comment 4:** **Regarding the Reviewer #2's comment on the capitalization of compound names, please keep them non-capitalized in the text and capitalized in Table 2.**
Response: In accordance with the journal’s instructions, compound names have been retained as lowercase in the main text and capitalized in Table 2.

**Comment 5:** **Note that PLOS ONE do not copyedit the text before publishing. Therefore, I suggest the authors having it thoroughly checked by a native English speaker or a professional editing agency to polish the language.**
Response: The manuscript has been carefully revised and edited to ensure clarity, correctness, and readability in English.

**[Note: HTML markup is below. Please do not edit.]

Reviewers' comments:

Reviewer's Responses to Questions**

**Comments to the Author

1. If the authors have adequately addressed your comments raised in a previous round of review and you feel that this manuscript is now acceptable for publication, you may indicate that here to bypass the “Comments to the Author” section, enter your conflict of interest statement in the “Confidential to Editor” section, and submit your "Accept" recommendation.**

**Reviewer #1: (No Response)**

**Reviewer #2: (No Response)**

**2. Is the manuscript technically sound, and do the data support the conclusions?

The manuscript must describe a technically sound piece of scientific research with data that supports the conclusions. Experiments must have been conducted rigorously, with appropriate controls, replication, and sample sizes. The conclusions must be drawn appropriately based on the data presented.**

**Reviewer #1: Yes**

**Reviewer #2: Yes**

**3. Has the statistical analysis been performed appropriately and rigorously?**

**Reviewer #1: Yes**

**Reviewer #2: I Don't Know**

**4. Have the authors made all data underlying the findings in their manuscript fully available?

The PLOS Data policy requires authors to make all data underlying the findings described in their manuscript fully available without restriction, with rare exception (please refer to the Data Availability Statement in the manuscript PDF file). The data should be provided as part of the manuscript or its supporting information, or deposited to a public repository. For example, in addition to summary statistics, the data points behind means, medians and variance measures should be available. If there are restrictions on publicly sharing data—e.g. participant privacy or use of data from a third party—those must be specified.**

**Reviewer #1: Yes**

**Reviewer #2: Yes**

**5. Is the manuscript presented in an intelligible fashion and written in standard English?

PLOS ONE does not copyedit accepted manuscripts, so the language in submitted articles must be clear, correct, and unambiguous. Any typographical or grammatical errors should be corrected at revision, so please note any specific errors here.**

**Reviewer #1: Yes**

**Reviewer #2: Yes**

**6. Review Comments to the Author

Please use the space provided to explain your answers to the questions above. You may also include additional comments for the author, including concerns about dual publication, research ethics, or publication ethics. (Please upload your review as an attachment if it exceeds 20,000 characters)**

**______________________________________________________________________________**

**Reviewer #1: i would like to acknowledge the effort of the authors in this paper, in order to improve the quality of this paper we recommand the application of the following remarks:

1. Please bring back the 'Amman 11733, Jordan' to the upper line.**

Response: The address has been corrected as requested **2. correct the abstract.**

Response: The abstract has been corrected as requested. **3. please check PLOS One guide for authors. the citations have to be between brackets [1], check the following link:** [**https://journals.plos.org/plosone/s/submission-guidelines#loc-references**](https://journals.plos.org/plosone/s/submission-guidelines#loc-references)**”**

Response: All references have been reformatted to the required style in accordance with PLOS ONE guidelines.

**4. the GC-MS method, some parameters are missing please add them they are as follow: the apparatus, the column, the oven temperature, the detector type and temperature, the vector gas, the flow rate...**

Response: Full GC-MS details (apparatus, column, oven temperature program, detector type and temperature, carrier gas, and flow rate) have been added to the Materials and Methods section (*2.3. Gas Chromatography-Mass Spectrometry).*

**5. please add the equation using the microsoft word equation insert; add number of equation (1), and cite it on the text (using the formula (1) below).**

Response: The equations 1 and 2 have been inserted using the MS Word equation editor, numbered accordingly and cited within the text.

**6. add reference of this method**

Response: Relevant references have been included.

**7. for each result please add how is it calculated (an equation or extrapolation from graph.**

Response: For each reported result, we have clarified how values were obtained, whether calculated by equation, extrapolated from graphs, or directly obtained from instruments.
 **8. the LC-MS analysis part is not complete, please add the following informations, which solvent is used to prepare the samples, add the type of column you used as well as its dimentions, you also must add the temperatures, the flowrate and the the elution system (whhich solvents, gradient or isocratic and the ratios), which system have you used for the injection, have you used internal standards ?**

Response: All the requested details regarding LC-MS have been included in the methods section *(2.8.* *LC-MS Analysis)*.

**9. down the tables add the abreviations.**

Response: Abbreviations have been added below each table where relevant **10. more details about monitoring the physical stability, is it with an apparatus, how is it quantified, and what is the principle of this method.**

Response: The physical stability monitoring method has been fully described, including apparatus, principles of measurement, and parameters observed
 **11. for all those calculations add the informations related to, which apparatus was used, how is the value calculated (equation, equation extrapolation or given by the apparatus), add also the unit of measurement.**

Response: Additional details regarding apparatus, calculation methods, and units have been added for clarity and reproducibility.

**12. in table 3, please center the cells content (Extract and TFC are up, when TPC is centered)**

Response: The alignment of cells in Table 3 has been corrected **13. in table 6, add MDS, PDI, ZP as abreviation down the table**

Response: Abbreviations (MDS, PDI, ZP) have been added below Table 7 in the new version. **Please check carefully the comments in the pdf file, Thank you**

Response: We have carefully reviewed and addressed all in-file comments and annotations in the attached PDF. The corresponding revisions have been implemented throughout the manuscript and summarized point-by-point in this letter. All edits are visible in the Revised Manuscript with Track Changes. This includes corrections to figure labels, table abbreviations, terminology consistency, and alignment of numerical values with the tables. The only exception is the comment regarding adding a reference next to each activity. Since several references were cited multiple times for different activities, we opted to avoid unnecessary repetition and instead maintained a clear, consolidated referencing style.

**______________________________________________________________________________**

**Reviewer #2: This study addresses an interesting and timely topic. The obtained results are significant and may benefit future research. However, before publishing this work, it is necessary to make certain corrections and clarify some doubts. I hope that my comments will contribute to improving the quality of the manuscript.**

**In the Abstract, you say: „Spontaneous emulsification was used to formulate ethanolic NEs, with S3, S4, S5, and S6 demonstrating thermodynamic stability, suitable pulmonary pH, and droplet sizes below 200 nm.”… but in the Material and Method section, you indicate that further analyses of thermodynamic stability were done on S4. Please additionally explain what was done and for which NE.**

Response: We thank the reviewer for pointing that out. The abstract, Materials and methods section, and Results section have been revised to describe the preparation of ethanol extract-loaded nanoemulsions (S1-S4 NEs) and identify the optimal S4 NE that underwent further analyses.

Spontaneous emulsification was used to formulate ethanolic nanoemulsions (S1-S4 NEs) with varying concentrations of ethanol extract, surfactant, cosurfactant, and oil phase. The optimal S4 NE demonstrated thermodynamic stability, appropriate pulmonary pH, and droplet sizes below 100 nm.

New section has been added (3.6. *Ethanol Extract-loaded Nanoemulsions)*

**Page 12, line 127: Indicate which cancer cell lines (lung cancer) were used in the study.**

Response: The cancer cell lines used in this study have been clearly specified in the revised Methods section; Human lung cancer cell lines A549, H1299.

**Page 12, line 136: Add the used concentration of MT reagent.**

Response: The concentration of the MTT reagent has been added to the Methods section; 15 μL (0.5mg/mL in each well) of MTT.

**Page 13, line 127:** **Add an explanation for S4 selection for thermodynamic studies.**

Response: We thank the reviewer for pointing that out. The following paragraph has been added to explain the selection of S4 NE for thermodynamic stability.

S4 NE was prepared by reducing the volume of oleic acid to 0.25 mL while keeping the other components constant (250 µg of *V. montana* ethanol extract, 2.25 mL of ethanol, 1.25 mL of Tween 20, and 1.25 mL of deionized water). S4 NE showed initial measurements of MDS, PDI, and ZP being 13.6 ± 0.5 nm, 0.1 ± 0.05, and -1.7 ± 0.6 mV, respectively. Its droplet size was within the acceptable range for NE (below 100 nm) [1]. Therefore, S4 NE was selected for further characterization and subjected to thermodynamic and physical stability studies.

This explanation has been added to the revised version of the manuscript.

**Page 13, line 165: Specify which physical characteristics are monitored.**

Response: We thank the reviewer for pointing that out. The physical characteristics, including phase separation, homogeneity, and color change, of the optimal S4 NE have been monitored visually and added to the revised manuscript. The following sections has been modified as follows:

2.10.5. Physical Stability

Physical stability of *C. montanus* NEs, including phase separation, homogeneity, and color change, was monitored visually for 60 days at RT and at 4 °C.

3.7.5. Physical Stability

The physical evaluation of S4 NE after two months of storage at RT and 4 °C revealed that the nanoemulsion remained transparent and uniform with no color change or phase separation.

**In the Materials and Methods, rotate the subheadings 2.11.2 and 2.11.3 so that it is displayed the same as in the Results (or do the rotation in the Results).**

Response: We thank the reviewer for pointing that out. The subheadings 2.11.2 and 2.11.3 have been rotated in the Methods and Results Sections.

**Page 14, line 185: „hydrocarbon monoterpenes (37.7%) and oxygenated monoterpenes (36.9%) as shown in Table 2.”… There is a mistake here or in the table regarding the values.**

Response: Consistencies between text and Table 2 values have been corrected.

**Page 14, line 193: “1,8-cineole (35.60%), sabinene (12.70%), and α-artemisia triene (11.40%). “…Is it necessary to capitalise compound names as written in Table 2? If so, implement the changes throughout the entire manuscript.**

Response: Compound names are lowercase in the text and capitalized in Table 2, following journal guidelines**.**

**Same line: “1,8-cineole (35.60%)” …value does not correspond to value from Table 2.**

Response: Consistencies between text and Table 2 values have been corrected.

**In the text of the manuscript, you use the term aqueous extract (Page 16, line 202), but in the tables and figures, you use the term water extract. Use one term and make the appropriate changes. Also, the order in which extracts are displayed is not uniform in tables and graphs. Please correct it. Apply the selected term and order of extracts to the figures, also.**

Response: The terminology has been standardized across the text, tables, and figures, using “water extract.” The order of extracts has also been made uniform.

**Results for Antioxidant activity are not displayed.**

Response: We respectfully note that the results on antioxidant activity were originally presented in Section 3.3 (Antioxidant Activity) in textual form. In the revised version, these results have been consolidated into new table (Table 4) and are further elaborated upon in both the Results and Discussion sections.

**The plant name remained unchanged on the figures.**

Response: The plant name has been corrected and updated consistently across all figures.

**The Discussion section should be expanded by comparing the obtained results with the literature data, as well as emphasising the significance of the results of this work. For example, the results of NE cytotoxicity indicate a significantly reduced IC_50_ value of the used NE compared to the corresponding extract, but this is not emphasised or interpreted anywhere in the work.**

Response: We thank the reviewer for pointing that out. The Discussion section has been expanded by comparing the obtained results with the literature data.

**Recent investigations are focused on *In vitro* callus induction, which generally allows for enhanced production of certain plant extract components, thereby contributing to their enhanced efficacy. It would be useful to mention this in combination with the spontaneous emulsification of the extract as a suggestion for future research. Here are some proposed references:**

**Abu-Darwish, D., Shibli, R., & Al-Abdallat, A. M. (2022). In vitro cultures and volatile organic compound production in chiliadenus montanus (vhal.) brullo. *Plants*, *11*(10), 1326.**

**Abu-Darwish, D., Shibli, R., & Al-Abdallat, A. M. (2024). Phenolic Compounds and Antioxidant Activity of Chiliadenus montanus (Vhal.) Brullo. grown in vitro. *Jordan Journal of Pharmaceutical Sciences*, *17*(3), 611-628.**

Response: A paragraph has been added to the Discussion suggesting in vitro callus induction and spontaneous emulsification of extracts as potential future research directions, and the recommended references have been cited (Abu-Darwish et al., 2022; Abu-Darwish et al., 2024).

**7. PLOS authors have the option to publish the peer review history of their article (what does this mean?). If published, this will include your full peer review and any attached files.


If you choose “no”, your identity will remain anonymous but your review may still be made public.


Do you want your identity to be public for this peer review? For information about this choice, including consent withdrawal, please see our Privacy Policy.**

**Reviewer #1: Yes: Hicham Mechqoq**

**Reviewer #2: No**

**[NOTE: If reviewer comments were submitted as an attachment file, they will be attached to this email and accessible via the submission site. Please log into your account, locate the manuscript record, and check for the action link "View Attachments". If this link does not appear, there are no attachment files.]

While revising your submission, please upload your figure files to the Preflight Analysis and Conversion Engine (PACE) digital diagnostic tool, https://pacev2.apexcovantage.com/. PACE helps ensure that figures meet PLOS requirements. To use PACE, you must first register as a user. Registration is free. Then, login and navigate to the UPLOAD tab, where you will find detailed instructions on how to use the tool. If you encounter any issues or have any questions when using PACE, please email PLOS at figures@plos.org. Please note that Supporting Information files do not need this step.**

Response: We appreciate the Editor’s guidance. All figure files have been uploaded to the PACE system as instructed, and they were checked to ensure compliance with PLOS formatting requirements.

______________________________________________________________________________

We have carefully addressed all comments and believe the revised manuscript has been significantly improved. We thank the reviewers and editor for their valuable suggestions, which helped strengthen our work.

Sincerely,
Nour Aboalhaija, PhD

Associate Professor of Phytochemistry

Department of Pharmacy

Faculty of Pharmacy

Al-Zaytoonah University of Jordan

Mobile: +962-79-6304081

E-mail: [n.aboalhaija@zuj.edu.jo](mailto:suhair.sunoqrot@zuj.edu.jo)

Google Scholar: <https://scholar.google.com/citations?user=CBVZdtcAAAAJ&hl=en>

ResearchGate: <https://www.researchgate.net/profile/Nour-Aboalhaija>
